# Electrochemically Induced, Metal Free Synthesis of 2‐substituted chroman‐4‐ones

**DOI:** 10.1002/open.202400395

**Published:** 2024-11-16

**Authors:** Mohsen Monirialamdari, Aleksandra Podlaska, Dominika Pomikło, Anna Albrecht

**Affiliations:** ^1^ Institute of Organic Chemistry Faculty of Chemistry Lodz University of Technology Żeromskiego 116 90-924 Łódź Poland; ^2^ Institute of General and Ecological Chemistry Faculty of Chemistry Lodz University of Technology Żeromskiego 116 90-924 Łódź Poland

**Keywords:** 2-substituted chromones, Decarboxylative alkylation, Decarboxylation process, Electrochemically induced Giese reaction, Redox-Active Esters (RAEs)

## Abstract

Electrochemically induced, decarboxylative functionalization of chromone‐3‐carboxylic acids by *N*‐hydroxyphthalimide esters as alkyl radical precursors was studied. Electrochemical protocol offers a sustainable and green approach, obviating the need for catalysts, relying on the direct reduction of NHPI esters using electric current. Developed protocol provides a straightforward route to the synthesis of diverse molecules with potential biological activity.

## Graphical abstract

This research presents a groundbreaking electrochemical method for the decarboxylative functionalization of chromone‐3‐carboxylic acids, utilizing *N*‐hydroxyphthalimide esters as radical precursors. This innovative approach provides a catalyst‐free, efficient pathway to synthesize a variety of 2‐substituted chroman‐4‐ones under mild conditions. This advancement not only broadens the scope of sustainable organic synthesis but also facilitates the production of biologically significant compounds.

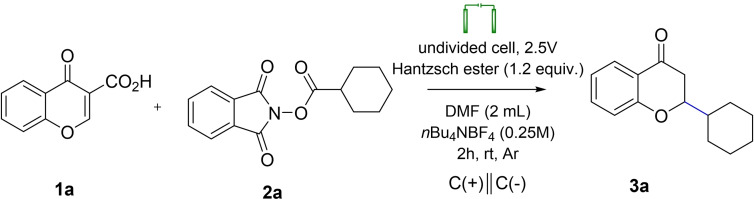

## Introduction

**Scheme 1:**
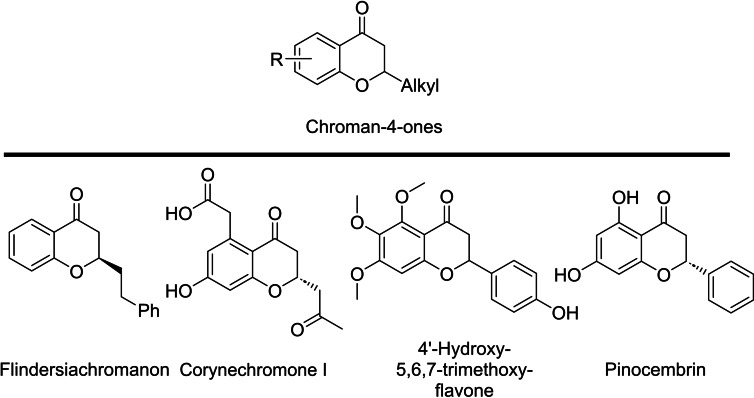
Naturally occurring chromanone derivatives‐representative examples.

Chromanones and related compounds, illustrated in Scheme 1, are crucial structural elements found in various natural products.^1^, ^2^ These compounds possess diverse biological activities, yet methods for their preparation remain limited.^[3]^ Flidersiachromon^[4]^ was isolated from bark of *Flindersia laevicarpa* and Corynechromone I derived from fungus *Corynespora cassiicola*.^[5]^ Flavanoids, such as Pinocembrin, are associated with reducing risk of certain chronic diseases.^[6]^ Natural flavanones isolated from flowers of *Chromolaena odorata* such as 4′‐hydroxy‐5,6,7‐trimethoxyflavanone are reported to have antimycobacterial activity.^[7]^

Emerging redox methodologies, propelled by advanced techniques like visible‐light photoredox strategies and electrosynthesis, are reshaping contemporary organic chemistry.^[8]^ These innovative approaches offer unique selectivities and exceptional tolerance towards functional groups, yet the conversion of key building blocks remains an ongoing challenge. Despite historical challenges in utilizing radicals due to hazardous reagents and harsh conditions,^[9]^ recent advancements in photoredox catalysis,^[10]^ electrochemistry,^[11]^ and transition‐metal catalysts^[12]^ have transformed their role as synthons in modern organic synthesis, offering complementary reactivity to polar pathways.^[13]^ Barton esters initially showed promise,^[14]^ their drawbacks, including thermal and photochemical instability, led to the emergence of *N*‐hydroxyphthalimide esters (NHPI) as convenient alternatives (Scheme 1a). NHPI esters, are known as “redox‐active esters” (RAEs), offering adaptability in response to their surroundings.^[15]^ In recent years, NHPI esters have found widespread use in photochemical synthesis.^[16]^ However development of reductive decarboxylation of NHPI esters has been relatively limited.^[17]^ In the literature Giese reaction is described as the addition of free radicals to electron‐deficient olefins.^[18]^ Photocatalytic decarboxylative Giese reactions leveraging photoredox catalysts and redox‐active systems have been described (Scheme 1b).^[19]^ These strategies enable efficient functionalization of activated olefins, yielding 2‐substituted chroman‐4‐ones or azauracils showcasing the potential of radical‐driven decarboxylation.^[19a,d]^ Recognizing electrochemistry's efficacy in bond formation, we have devised an electrochemical method for the decarboxylative C2 alkylation of chromone‐3‐carboxylic acids (Scheme 1c). This process employs *N*‐hydroxyphthalimide esters as alkyl radical precursors and chromone‐3‐carboxylic acids presenting a convenient route to synthesizing structurally privileged motifs with diverse biological activities.

**Scheme 2:**
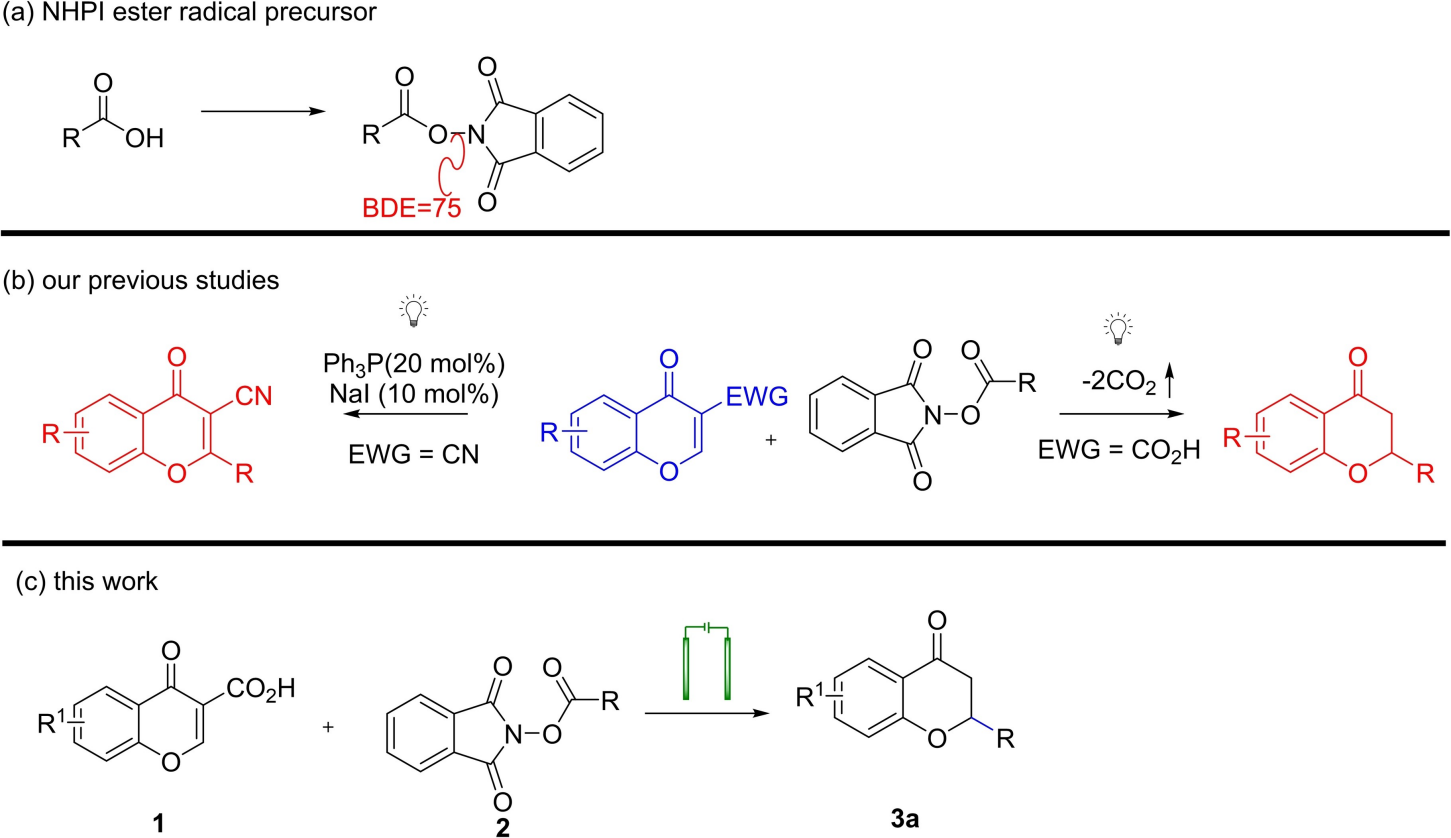
Electro‐Induced Giese Reaction‐the synthetic goal of our study.

Herein, we present our studies on the development of electrochemically induced, metal free synthesis of 2‐substituted chroman‐4‐ones. [Scheme 2].

## Results and Discussion

Optimization studies were began with the reductive decarboxylation of 1,3‐dioxoisoindolin‐2‐yl cyclohexane carboxylate **2 a** and subsequent Giese addition to chromone‐3‐carboxylic acid **1 a** (Table 1). The reaction was performed in *N,N*‐dimethylformamide in the presence of Hantzsch ester at room temperature under argon atmosphere employing electrochemical conditions (graphite electrodes, undivided cell, constant voltage 2.5 V) (Table 1, entry 1). Under these conditions **3 a** was produced with 32 % yield, therefore further optimization studies were performed (Table 1). Firstly, the voltage was raised to 5 V, however, the reaction was unsuccessful. Then, the influence of the reductant was checked (Table 1, entries 3–4). Reactions with γ‐terpinene as well as with 2‐fold excess of Hantzsch ester also resulted in obtaining product **3 a**, but with lower yields. The most successful was the use of 1.2‐fold excess of Hantzsch ester. The addition of Na_2_S_2_O_8_ acting as an exogenous oxidant (Table 1, entry 5) did not provide the expected outcome. The target product **3 a** was obtained with a similar yield to standard reaction conditions. In the course of further studies, different type of the electrolytes were evaluated. *n*Bu_4_NPF_6_ (Table 1, entry 6) and *n*Bu_4_NClO_4_ (Table 1, entry 7) did not result in obtaining the desired product. The experiment was successfully performed with different solvents. Aprotic solvents such as DCM and DME gave **3 a** in 33 % and 27 % yield, respectively (Table 1, entries 8–9). The best result was obtained using MeCN (Table 1, entry 10). Therefore further experiments were conducted using acetonitrile as a solvent. Replacement of graphite electrode (C) by reticulated vitreous carbon (RVC) and glassy carbon electrodes gave good, but similar results (Table 1, entry 11–12). A similar outcome was observed when the RCV graphite, Ni foam, and Pt electrodes were used as anodes (Table 1, entries 13–15). Changing the concentration of electrolyte from 0.25 to 0.15 and 0.20 (Table 1, entries 16–17) resulted in obtaining **3 a** with the yield of 43 % and 55 %, respectively. Open air conditions (Table 1, entry 18) drastically lowered the yield. Notably, the optimized reaction is inhibited when performed without reductant and current (Table 1, entry 19 and 20). The quenching experiment with TEMPO was also carried out confirming the radical nature of the developed protocol (Table 1, entry 21). Experiment with reference electrode was performed and the potential remains constant (Table 1, entry 22).

**Table 1:** Electro‐Induced Giese Reaction‐optimization studies.

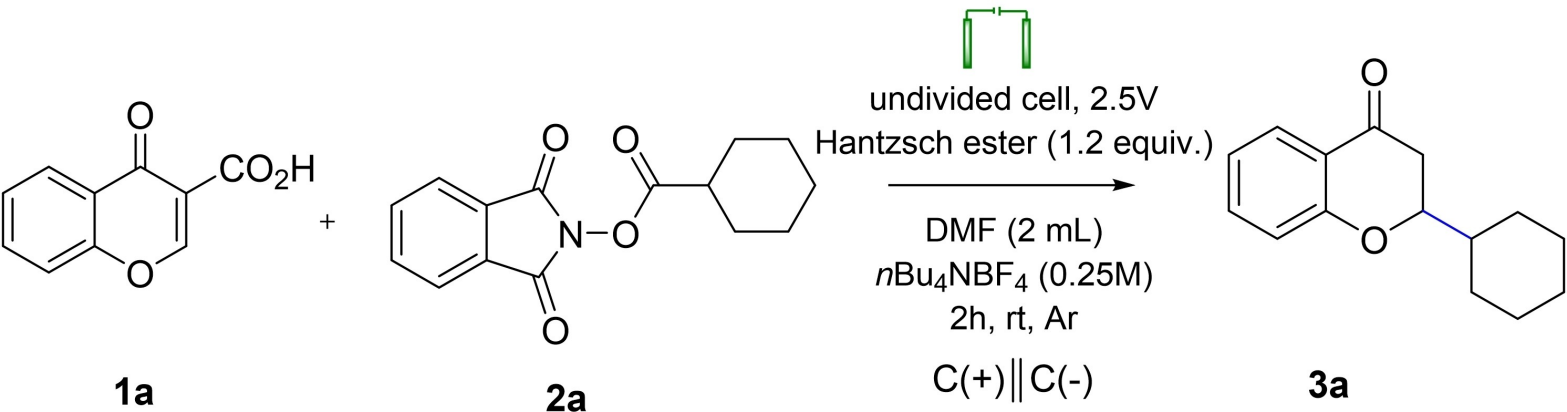		
Entry	Deviation from standard conditions	Yield [%]
	None	32
2	Constant voltage (5 V)	‐
3	γ‐Terpinene as a reductant	24
4	Hantzsch ester (2 eqiuv.)	19
5	Exogenous oxidant (Na_2_S_2_O_8_)	36
6	*n*Bu_4_NPF_6_ as electrolyte	‐
7	*n*Bu_4_NClO_4_ as electrolyte	‐
8	CH_2_Cl_2_ as a solvent	33
9	1,2‐Dimethoxyethane as a solvent	27
10	CH_3_CN as a solvent	70
11	RVC foam electrodes in CH_3_CN	38
12	Glassy carbon electrodes in CH_3_CN	33
13	RVC (+)|graphite (−) in CH_3_CN	48
14	RVC (+)|Ni foam (−) in CH_3_CN	49
15	RVC (+)|Pt (−) in CH_3_CN	53
16	0.15 M *n*Bu_4_NBF_4_ in CH_3_CN	43
17	0.2 M *n*Bu_4_NBF_4_ in CH_3_CN	55
18	Under open air conditions	11
19	No reductant	‐
20	No current	‐
21	TEMPO	‐
22	With reference electrode	70

Standard conditions: **1 a** (0.15 mmol, 1.0 equiv.) and **2 a** (2.0 equiv.) in the presence of the Hantzsch ester (1.2 equiv.) and *n*Bu_4_NBF_4_ (0.5 mmol, 0.25 M) in the DMF (2 mL) graphite electrodes were used, on an IKA ElectraSyn 2.0 stir plate and electrolysis was set to 2.5 V, 2 h at room temperature in Argon atmosphere.

**Scheme 3:**
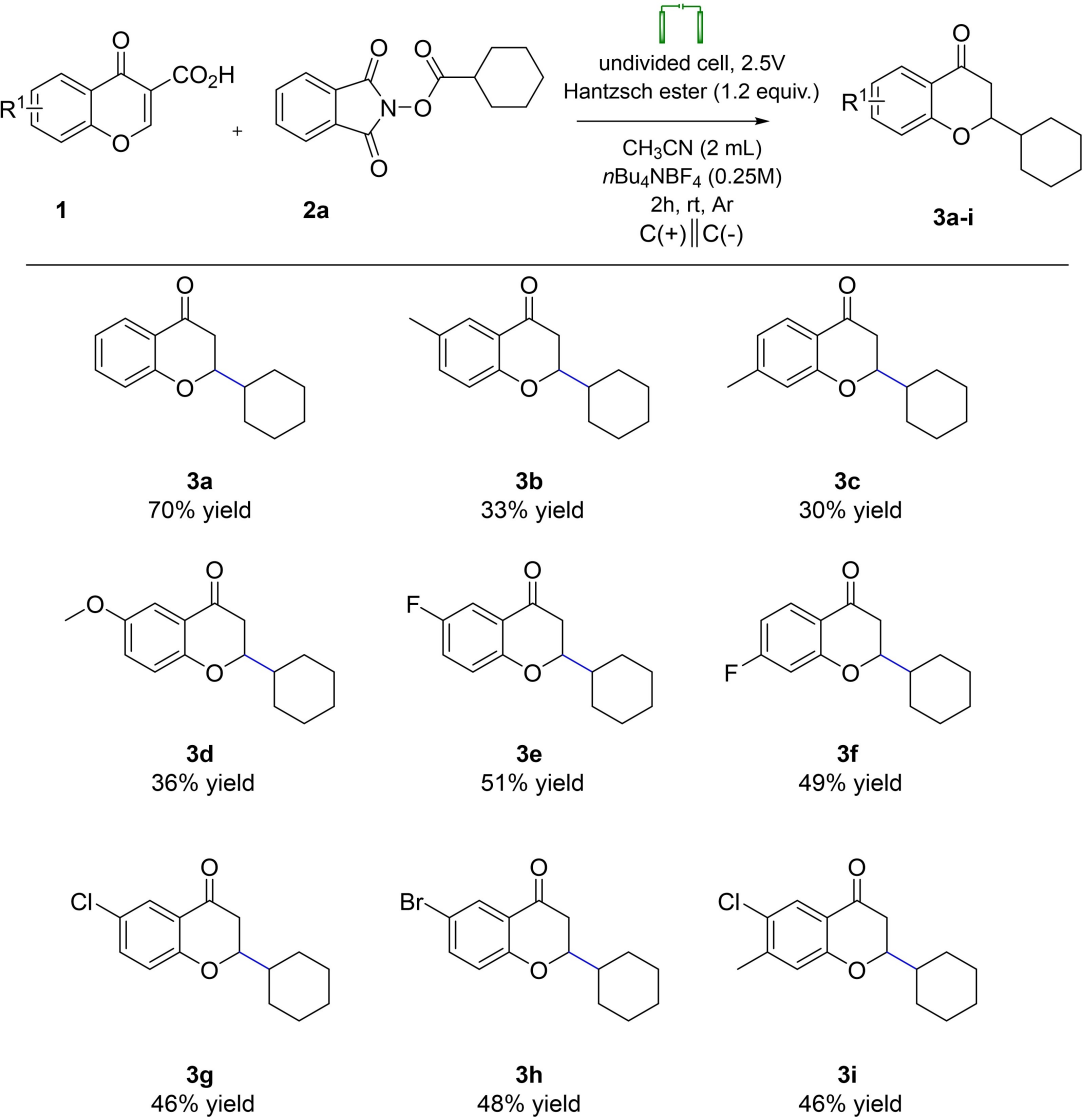
Scope of Chromone‐3‐Carboxylic Acids in Electro‐Induced Giese Reaction.

**Scheme 4:**
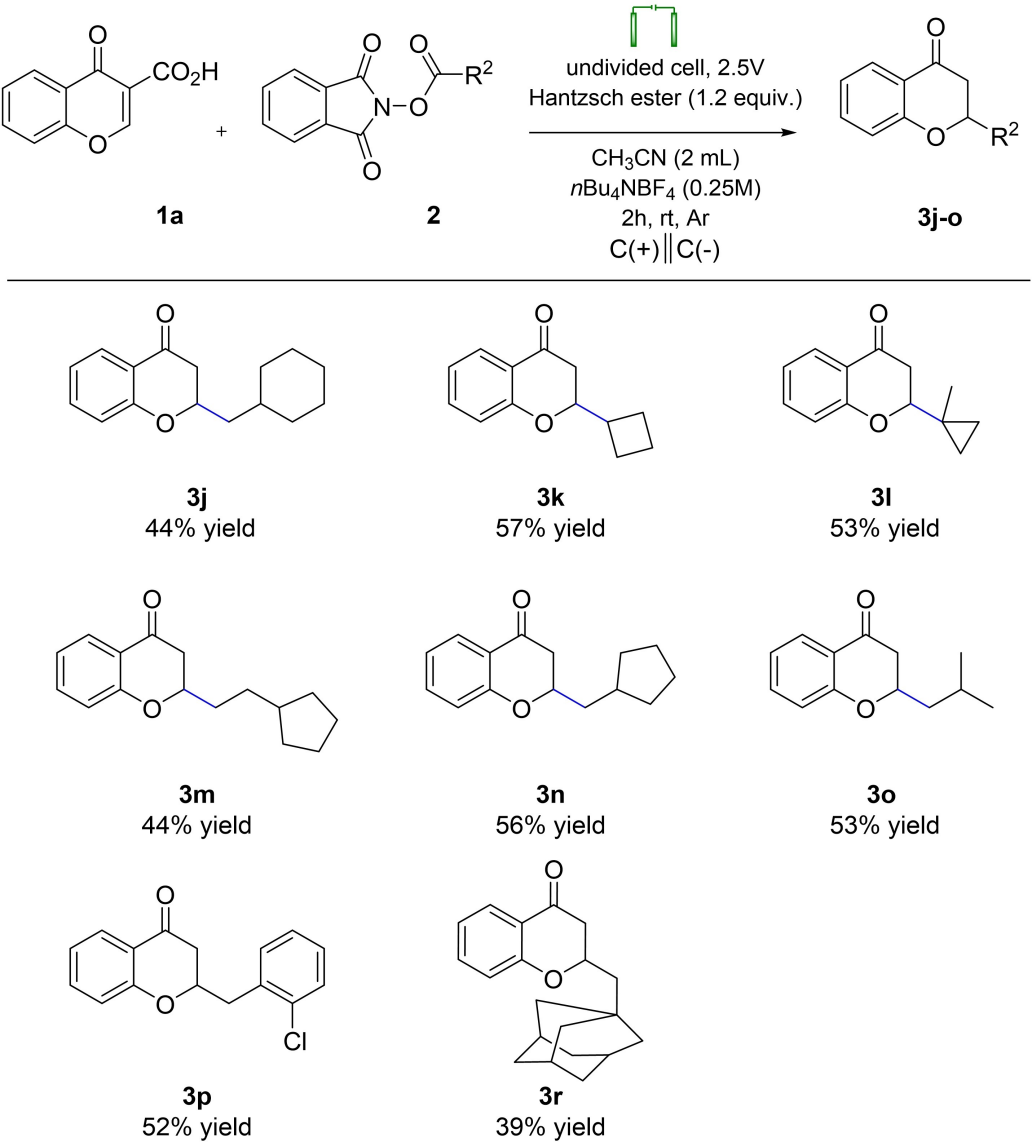
Scope of Redox‐Active Esters (RAEs) in Electro‐Induced Giese Reaction.

Having accomplished the optimization studies, the goal of establishing scope and limitation of the methodology was pursued (Schemes 3 and 4). Initially, chromone‐3‐carboxylic acids **1** were tested. They were categorized based on their electronic properties: (1) featuring electron‐donating groups on the aromatic ring, (2) featuring electron‐withdrawing groups on the aromatic ring, and (3) featuring opposite electronic effects on the aromatic ring as illustrated in the Scheme 3. Chromone‐3‐carboxylic acids **1** bearing electron‐donating groups smoothly underwent the reaction under electrochemical conditions, yielding the desired products **3 b**‐**d** in modest yields (33 %, 30 %, and 36 % yield for 6‐Me (**3 b**), 7‐Me (**3 c**), and 6‐methoxy (**3 d**) derivatives, respectively). Chromone‐3‐carboxylic acids containing electron‐withdrawing groups also exhibited good compatibility, producing the desired products in notable yields (51 %, 49 %, 46 %, and 48 % yield for 6‐fluoro (**3 e**), 7‐fluoro (**3 f**), 6‐chloro (**3 g**), and 6‐bromo (**3 h**) derivatives, respectively), thus showcasing broad functional group tolerance. Moreover, chromone‐3‐carboxylic acid featuring substituents of opposite electronic effects, such as 6‐chloro‐7‐methyl derivative (**3 i**), yielded the corresponding products in moderate yields (46 %). However, it should be noted that nitro‐ and hydroxy‐substituted chromone‐3‐carboxylic acids at the 6‐position of the aromatic ring failed to yield the desired product under the catalyst‐free and mild electrochemical conditions.

In the subsequent phase of our investigation, the compatibility of primary, secondary, and tertiary Redox‐Active Esters (RAEs) harboring diverse functional groups under optimized electrochemical conditions was evaluated (Scheme 4). This methodology afforded synthetically valuable products (designated as **3 j**–**3 r**) with yields ranging from 39 % to 57 %, obviating the necessity for transition metal catalysts. Significantly, this approach demonstrated its efficacy for both cyclic and acyclic radicals, further underscoring its versatility and applicability in organic synthesis.

**Scheme 5:**
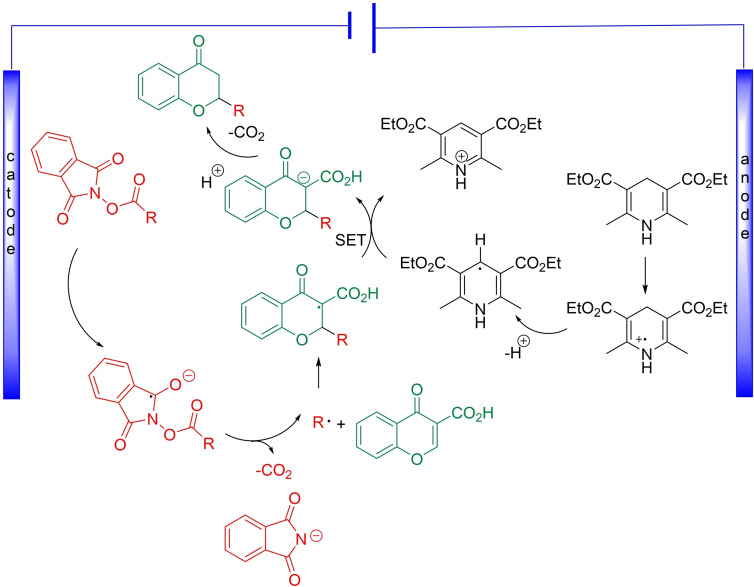
Mechanism of Electro‐Induced Giese Reaction.

In the course of further studies, plausible mechanism for the decarboxylative electrochemically induced alkylation of chromone‐3‐carboxylic acids **1** was proposed (Scheme 5). The process is initiated by cathodic reduction of *N*‐(acyloxy)phthalimide leading to decarboxylative formation of the corresponding radical.^[17b]^ The newly regenerated radical participates in the Giese‐type addition to chromone‐3‐carboxylic acids to give a new radical. At the same time Hantzsch ester undergoes anodic oxidation to give radical cation that upon deprotonation forms a strongly reducing radical. The single electron transfer (SET) between two radicals leads to the formation of anion. Its protonation completes the cycle providing a target product **3**.

## Conclusions

In conclusion, we have developed an electrochemically induced, metal free synthesis of 2‐substituted chroman‐4‐ones **3** that constitute a unique application of activated olefins in radical transformations. Developed protocol was performed under electrochemical conditions between chromone‐3‐carboxylic acids **1** and *N*‐(acyloxy)phthalimides **2** in the presence of diethyl 1,4‐dihydro‐2,6‐dimethyl‐3,5‐pyridinedicarboxylate as a reductant at room temperature for 2 h. The methodology proved versatile leading to biologically relevant 2‐substituted‐chromones **3** in good yields under mild reaction conditions without external oxidant.

## Experimental Section

### General Procedure for the Synthesis of 3 a‐r

In the 10 mL IKA vial, chromone‐3‐carboxylic acid **1 a‐i** (0.15 mmol, 1.0 equiv), *N*‐(acyloxy)phthalimide **2** (0.3 mmol, 2.0 equiv), Hantzsch ester (0.18 mmol, 45.6 mg), *n*BuNBF_4_ (0.5 mmol, 164.6 mg) were dissolved in dry CH_3_CN (2 mL). Vial cap was equipped with graphite electrodes. Reaction mixture was degassed and filled three times with argon. Subsequently, the mixture was then placed on an IKA ElectraSyn 2.0 stir plate and electrolysis was set to 2.5 V, reaction was provided for 2 h at room temperature. Next, the reaction was quenched with water (10 mL), extracted with ethyl acetate (3×10 mL) and washed with brine (5 mL). The organic phase was dried over Na_2_SO_4_ and concentrated under reduced pressure. The crude product was purified by silica gel chromatography (*n*‐hexane:ethyl acetate 20 : 1) to provide the desired products **3 a‐r**.

## Conflict of Interests

The authors declare no conflict of interest.

## Supporting information

As a service to our authors and readers, this journal provides supporting information supplied by the authors. Such materials are peer reviewed and may be re‐organized for online delivery, but are not copy‐edited or typeset. Technical support issues arising from supporting information (other than missing files) should be addressed to the authors.

Supporting Information

## Data Availability

The data that support the findings of this study are available from the corresponding author upon reasonable request.
