# Supplementary material for: Electrochemically Induced, Metal Free Synthesis of 2‐substituted chroman‐4‐ones
Source: ChemistryOpen. 2024 Nov 16;14(6):e202400395. doi: 10.1002/open.202400395 (PMC13062925; doi:10.1002/open.202400395)

ChemistryOpen

Supporting Information

Electrochemically Induced, Metal Free Synthesis of 2-substituted chroman-4-ones

Mohsen Monirialamdari, Aleksandra Podlaska, Dominika Pomikło, and Anna Albrecht*

Supplementary Information

Electrochemically induced, metal free synthesis of 2-substituted chroman-4-ones

Mohsen Monirialamdari,[a]. Aleksandra Podlaska[a] Dominika Pomikło[a] and Anna Albrecht[b*]

[a] Institute of Organic Chemistry, Department of Chemistry, Lodz University of Technology, Żeromskiego 116, 90-924 Łódź, Poland

[b] Institute of General and Ecological Chemistry, Department of Chemistry, Lodz University of Technology, Żeromskiego 116, 90-924 Łódź, Poland

anna.albrecht@p.lodz.pl

Contents

1. General information S2
2. Cyclic voltammetry S4
3. General procedure for the synthesis of 2-substituted-chroman-4-ones **3a-o** S5
4. NMR data S11

1. General information

NMR spectra were acquired on a Bruker Ultra Shield 700 instrument, running at 700 MHz for ^{1}H and 176 MHz for ^{13}C, respectively. Chemical shifts (δ) are reported in ppm relative to residual solvent signals ($CDCl_3$: 7.26 ppm for ^{1}H NMR, 77.16 ppm for ^{13}C NMR Mass spectra were recorded on a Bruker Maxis Impact spectrometer using electrospray (ES+) ionization (referenced to the mass of the charged species). Analytical thin layer chromatography (TLC) was performed using pre-coated aluminum-backed plates (Merck Kieselgel 60 F254) and visualized by ultraviolet irradiation. Unless otherwise noted, analytical grade solvents and commercially available reagents were used without further purification. For flash chromatography (FC) silica gel (Silica gel 60, 230-400 mesh, Fluka). Electrochemical equipment was purchased from commercial supplier IKA company. Chromone-3-carboxylic acids[1] **1** were synthetized according to the literature procedure. *N*-(Acyloxy)phthalimides (NHPI) **2** were prepared from the corresponding starting materials following the literature procedure.[2]

[1] Ishizuka, N.; Matsumura, K.; Sakai, K.; Fujimoto, M.; Mihara, S.; Yamamori, T. *J. Med. Chem.*, **2002**, *45*, 2041.
[2] Fawcett, A.; Pradeilles, J.; Wang, Y.; Mutsuga, T.; Myers, E. L.; Aggarwal, V. K. *Science*, **2017**, *357*, 283.

2. Cyclic voltammetry

Electrochemical characterization of redox active esters **2a**, **2j**, **2l** was conducted using a potentiostat (ElectraSyn 2.0, purchased from IKA) instrument. Compounds **2a**, **2j**, **2l** was dissolved in dry, spectroscopic grade DMF (concentration 1.0 mM) in the presence of $[n\text{-Bu}_4\text{N}]^+[\text{BF}_4]^-$ as an electrolyte (concentration 100 mM) and the resulting solution was degassed by purging with Ar gas for 25 minutes. A three-electrode electrochemical cell was used with Pt plated electrodes as the working and counter electrodes and Ag/AgCl (internal solution, 3.0 M KCl) as the reference electrode. Cyclic voltammetry (CV) measurements were started from 0.0 V in the reductive direction and conducted with scan rate 30 mV s^{-1}.

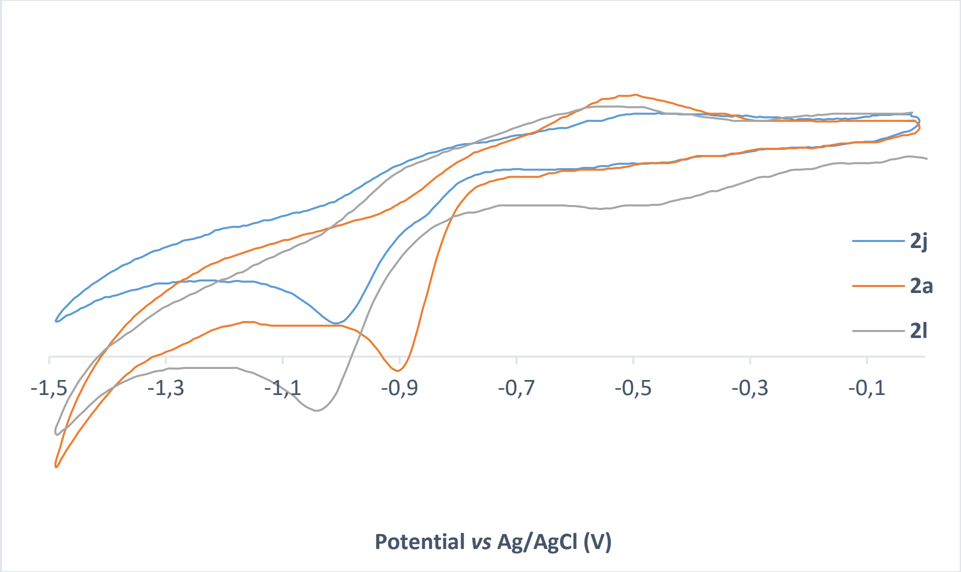

Fig. S1. Cyclic voltammograms recorded in a solution of **2a, 2j, 2l** and supporting electrolyte in DMF at the scan rate of 30 mV s^{-1}.

3. General procedure for the synthesis of 2-substituted-chroman-4-ones 3a-o

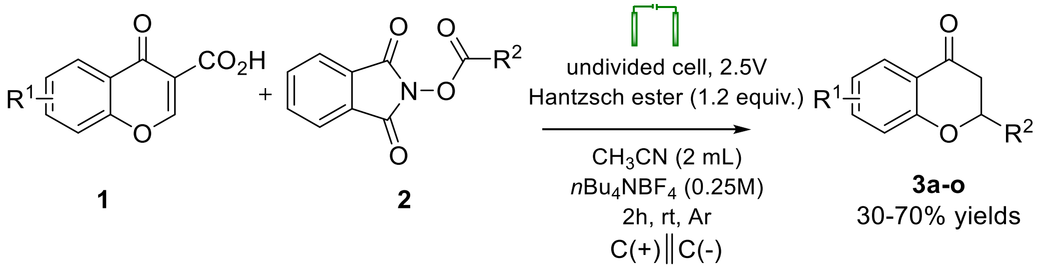

In the 10 mL IKA vial, chromone-3-carboxylic acid **1a-i** (0.15 mmol, 1.0 equiv), *N*-(acyloxy)phthalimide **2** (0.3 mmol, 2.0 equiv), Hantzsch ester (0.18 mmol, 45.6 mg), *n*$BuNBF_4$ (0.5 mmol, 164.6 mg) were dissolved in dry CH_3CN (2 mL). Vial cap was equipped with graphite electrodes. Reaction mixture was degassed and filled three times with argon. Subsequently, the mixture was then placed on an IKA ElectraSyn 2.0 stir plate and electrolysis was set to 2.5 V, reaction was provided for 2 h at room temperature. Next, the reaction was quenched with water (10 mL), extracted with ethyl acetate (3×10 mL) and washed with brine (5 mL). The organic phase was dried over Na_2SO_4 and concentrated under reduced pressure. The crude product was purified by silica gel chromatography (*n*-hexane:ethyl acetate 20:1) to provide the desired products **3a-o.**

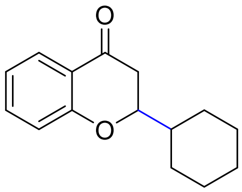

2-cyclohexylchroman-4-one (3a): Synthesized according to general procedure, yielding the desired product as yellow oil, 24 mg, yield: 70 %; Elutes with 5% EtOAc in petroleum ether.

^{1}H NMR (700 MHz, $CDCl_3$) δ 7.87 (dd, *J* = 7.8, 1.6 Hz, 1H), 7.44 – 7.48 (m, 1H), 6.96 – 7.00 (m, 2H), 4.20 (ddd, *J* = 12.9, 6.0, 2.9 Hz, 1H), 2.70 – 2.75 (m, *J* = 16.6, 12.9 Hz, 1H), 2.66 (dd, *J* = 16.6, 2.9 Hz, 1H), 1.82 (dd, *J* = 9.8, 3.2 Hz, 2H), 1.78 (d, *J* = 12.5 Hz, 1H), 1.70 – 1.76 (m, 2H), 1.27 – 1.36 (m, 3H), 1.13 – 1.24 (m, 3H). ^{13}C NMR (176 MHz, $CDCl_3$) δ 193.3, 162.1, 136.0, 127.1, 121.2, 121.2, 118.0, 82.2, 42.0, 40.4, 28.4, 28.4, 26.5, 26.1, 26.1.

HRMS (ESI+) m/z calcd. for $C_{15}H_{19}O_2^+$ $[M+H]^+$ 231.1379 found 231.1381.

The characteristics data is consistent with the one reported before. [3]

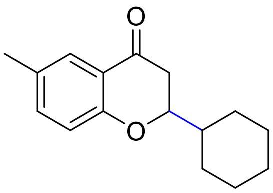

2-cyclohexyl-6-methylchroman-4-one (3b): Synthesized according to general procedure, yielding the desired product as yellow oil, 12 mg, yield: 33 %; Elutes with 5% EtOAc in petroleum ether.

^{1}H NMR (700 MHz, $CDCl_3$) δ 7.65 (d, *J* = 2.3 Hz, 1H), 7.27 (d, *J* = 8.1 Hz, 1H), 6.87 (d, *J* = 8.4 Hz, 1H), 4.16 (dd, *J* = 6.4, 4.4 Hz, 1H), 2.70 (dd, *J* = 16.6, 12.4 Hz, 1H), 2.63 (dd, *J* = 16.6, 3.6 Hz, 1H), 2.29 (s, 3H), 1.99 (d, *J* = 11.9 Hz, 1H), 1.68 – 1.86 (m, 5H), 1.11 – 1.31 (m, 5H). ^{13}C NMR (176 MHz, $CDCl_3$) δ 194.23, 160.88, 137.86, 131.27, 127.33, 121.52, 118.56, 82.83,42.68, 41.19, 29.20, 29.10, 27.24, 26.87, 26.81, 21.29.

HRMS (ESI+) m/z calcd. for $C_{16}H_{21}O_2^+$ [M+H]$^+$ 245.1536 found 245.1536.
The characteristics data is consistent with the one reported before. [3]

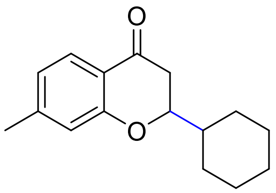

2-cyclohexyl-7-methylchroman-4-one (3c): Synthesized according to general procedure, yielding the desired product as yellow oil, 11 mg, yield: 30 %; Elutes with 5% EtOAc in petroleum ether.
^{1}H NMR (700 MHz, $CDCl_3$) δ 7.75 (d, *J* = 8.0 Hz, 1H), 6.79 – 6.81 (m, 1H), 6.77 – 6.78 (m, 1H), 4.17 (ddd, *J* = 12.9, 6.1, 3.0 Hz, 1H), 2.69 (dd, *J* = 16.6, 12.9 Hz, 1H), 2.62 (dd, *J* = 16.6, 3.0 Hz, 1H), 2.34 (s, 3H), 1.98 (d, *J* = 12.8 Hz, 1H), 1.71 – 1.81 (m, 5H), 1.11 – 1.31 (m, 5H). ^{13}C NMR (176 MHz, $CDCl_3$) δ 192.97, 162.08, 147.51, 126.92, 122.56, 118.95, 118.02, 82.12, 41.92, 40.36, 28.42, 28.38, 26.48, 26.10, 26.04, 22.04.
HRMS (ESI+) m/z calcd. for $C_{16}H_{21}O_2^+$ [M+H]$^+$ 245.1536 found 245.1536.
The characteristics data is consistent with the one reported before. [3]

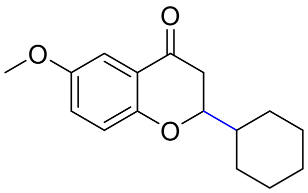

2-cyclohexyl-6-methoxychroman-4-one (3d): Synthesized according to general procedure, yielding the desired product as yellow oil, 14 mg, yield: 36 %; Elutes with 5% EtOAc in petroleum ether.
^{1}H NMR (700 MHz, $CDCl_3$) δ 7.29 (d, *J* = 3.2 Hz, 1H), 7.08 (dd, *J* = 9.0, 3.2 Hz, 1H), 6.90 (d, *J* = 9.0 Hz, 1H), 4.15 (ddd, *J* = 13.0, 6.1, 3.1 Hz, 1H), 3.79 (s, 3H), 2.70 (dd, *J* = 16.7, 13.0 Hz, 1H), 2.63 (dd, *J* = 16.7, 3.1 Hz, 1H), 1.98 (d, *J* = 13.0, Hz, 1H), 1.71 – 1.81 (m, 5H), 1.11 – 1.31 (m, 5H). ^{13}C NMR (176 MHz, $CDCl_3$) δ 193.37, 156.84, 154.00, 125.32, 120.93, 119.33, 107.37, 82.25, 55.94, 41.93, 40.34, 28.46, 28.37, 26.48, 26.11, 26.05.
HRMS (ESI+) m/z calcd. for $C_{16}H_{21}O_3^+$ [M+H]$^+$ 261.1485 found 261.1485.
The characteristics data is consistent with the one reported before. [4]

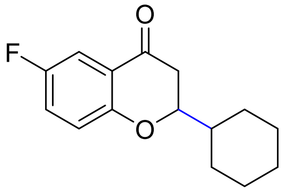

2-cyclohexyl-6-fluorochroman-4-one (3e): Synthesized according to general procedure, yielding the desired product as yellow oil, 19 mg, yield: 51 %; Elutes with 5% EtOAc in petroleum ether.
^{1}H NMR (700 MHz, $CDCl_3$) δ 7.51 (dd, *J* = 8.3, 3.2 Hz, 1H), 7.18 (ddd,*J* = 9.0, 7.7, 3.2 Hz, 1H), 6.95 (dd, *J* = 9.1, 4.2 Hz, 1H), 4.18 (ddd, *J* = 11.7, 5.9, 4.2 Hz, 1H), 2.71 (dd, *J* = 16.7, 12.6 Hz, 1H), 2.66 (dd, *J* = 16.7, 3.3 Hz, 1H) , 1.97 (d, *J* = 12.8 Hz, 1H), 1.72 – 1.80 (m, 5H), 1.12 – 1.31 (m, 5H). ^{13}C NMR (176 MHz, $CDCl_3$) δ 192.52, 158.33, 157.20 (d, *J* = 241.4 Hz), 123.57 (d, *J* = 24.6 Hz), 121.53 (d, *J* = 6.5 Hz), 119.67 (d, *J* = 7.3 Hz), 111.96 (d, *J* = 23.2 Hz), 82.42, 41.88, 40.16, 28.39, 28.35, 26.44, 26.07, 26.02. ^{19}F NMR (376 MHz, $CDCl_3$) δ -121.87 (td, *J* = 7.6, 4.2 Hz).
HRMS (ESI+) m/z calcd. for $C_{15}H_{18}FO_2^+$ [M+H]$^+$ 249.1285 found 249.1285.
The characteristics data is consistent with the one reported before. [3]

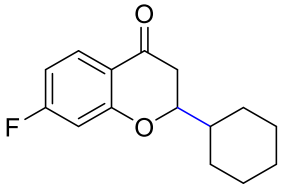

2-cyclohexyl-7-fluorochroman-4-one (3f): Synthesized according to general procedure, yielding the desired product as yellow oil, 18 mg, yield: 49 %; Elutes with 5% EtOAc in petroleum ether.

^{1}H NMR (700 MHz, $CDCl_3$) δ 7.88 (dd, *J* = 8.8, 6.7 Hz, 1H), 6.71 (td, *J*= 8.5, 2.4 Hz, 1H), 6.66 (dd, *J* = 9.9, 2.4 Hz, 1H), 4.22 (ddd, *J* = 12.3, 5.9, 3.6 Hz, 1H), 2.71 (dd, *J* = 16.6, 12.8 Hz, 1H), 2.64 (dd, *J* = 16.6, 3.1 Hz, 1H), 1.97 (dtt, *J* = 13.1, 3.4, 1.6 Hz, 1H), 1.69 – 1.87 (m, 5H), 1.12 – 1.31 (m, 5H). ^{13}C NMR (176 MHz, $CDCl_3$) δ 191.80, 167.64 (d, *J* = 255.5 Hz), 163.72 (d, *J* = 13.8 Hz), 129.56 (d, *J* = 11.5 Hz),118.14, 109.63 (d, *J* = 22.8 Hz), 104.76 (d, *J* = 24.2 Hz), 82.81, 41.85, 40.08, 28.35, 28.33, 26.43, 26.06,26.00.
^{19}F NMR (376 MHz, $CDCl_3$) δ -100.78(q, *J* = 7.8 Hz).
HRMS (ESI+) m/z calcd. for $C_{15}H_{18}FO_2^+$ $[M+H]^+$ 249.1285 found 249.1285.
The characteristics data is consistent with the one reported before. [3]

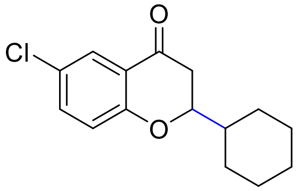

6-chloro-2-cyclohexylchroman-4-one (3g): Synthesized according to general procedure, yielding the desired product as yellow oil, 18 mg, yield: 46 %; Elutes with 5% EtOAc in petroleum ether.

^{1}H NMR (700 MHz, $CDCl_3$) δ 7.82 (d, *J* = 2.7 Hz, 1H), 7.40 (dd, *J* = 8.8,2.7 Hz, 1H), 6.93 (d, *J* = 8.8 Hz, 1H), 4.19 (ddd, *J* = 11.8, 5.9, 4.2 Hz, 1H), 2.71 (dd, *J* = 16.7, 12.5 Hz, 1H), 2.66 (dd, *J* = 16.7, 3.4 Hz, 1H) , 1.97 (d, *J* = 13.0 Hz, 1H), 1.70 – 1.82 (m, 5H), 1.12 – 1.31 (m, 5H). ^{13}C NMR (176 MHz, $CDCl_3$) δ 192.11,160.48, 135.88, 126.72, 126.39, 121.95, 119.79, 82.42, 41.84, 40.10, 28.36, 28.34, 26.42, 26.06, 26.00.
HRMS (ESI+) m/z calcd. for $C_{15}H_{18}ClO_2^+$ $[M+H]^+$ 265.0990 found 265.0990.
The characteristics data is consistent with the one reported before. [3]

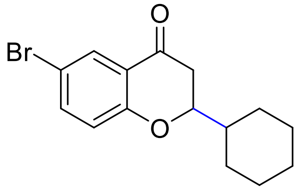

6-bromo-2-cyclohexylchroman-4-one (3h): Synthesized according to general procedure, yielding the desired product as yellow oil, 22 mg, yield: 48 %; Elutes with 5% EtOAc in petroleum ether.

^{1}H NMR (700 MHz, $CDCl_3$) δ 7.97 (d, *J* = 2.5 Hz, 1H), 7.53 (dd, *J* = 8.8, 2.5 Hz, 1H), 6.87 (d, *J* = 8.8 Hz, 1H), 4.19 (ddd, *J* = 11.5, 5.8, 4.2 Hz, 1H), 2.62 – 2.76 (m, 2H), 1.96 (d, *J* = 13.1 Hz, 1H), 1.72 – 1.86 (m, 5H), 1.23 – 1.32 (m, 5H). ^{13}C NMR (176 MHz, $CDCl_3$) δ 191.81, 161.92, 139.60, 129.40, 123.42, 121.14, 114.83, 82.23, 41.84, 40.06, 28.35, 28.33, 26.42, 26.05, 25.99.
HRMS (ESI+) m/z calcd. for $C_{15}H_{18}BrO_2^+$ $[M+H]^+$ 309.0485 found 309.0485.
The characteristics data is consistent with the one reported before. [3]

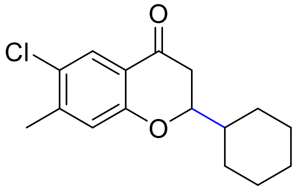

6-chloro-2-cyclohexyl-7-methylchroman-4-one(3i): Synthesized according to general procedure, yielding the desired product as yellow oil, 19 mg, yield: 46 %; Elutes with 5% EtOAc in petroleum ether.

^{1}H NMR (700 MHz, $CDCl_3$) δ 7.80 (d, *J* = 5.2 Hz, 1H), 6.87 (d, *J* = 0.5 Hz, 1H), 4.16 (ddd, *J* = 12.5, 6.1, 3.2 Hz, 1H), 2.68 (dd, *J* = 16.7, 12.6 Hz, 1H), 2.63 (dd, *J* = 16.7, 3.3 Hz, 1H), 2.37 (s, 3H), 1.96 (ddt, *J* = 12.7, 3.6, 1.8 Hz, 1H), 1.68 – 1.84 (m, 5H), 1.11 – 1.29 (m, 5H). ^{13}C NMR (176 MHz, $CDCl_3$) δ 191.98, 160.31, 145.03, 127.42, 126.73, 120.20, 120.13, 82.38, 41.85, 40.12, 28.37 (2xC), 26.44, 26.07, 26.00, 20.94.
HRMS (ESI+) m/z calcd. for $C_{16}H_{20}ClO_2^+$ [M+H]$^+$ 279.1146 found 279.1144 .
The characteristics data is consistent with the one reported before. [3]

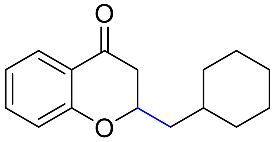

2-(cyclohexylmethyl)chroman-4-one (3j): Synthesized according to general procedure, yielding the desired product as yellow oil, 17 mg, yield: 44 %; Elutes with 5% EtOAc in petroleum ether.

^{1}H NMR (700 MHz, $CDCl_3$) δ 7.87 (ddd, *J* = 7.7, 1.8, 0.5 Hz, 1H), 7.47 (ddd, *J* = 8.3, 7.2, 1.8 Hz, 1H), 7.00 (ddd, *J* = 8.0, 7.2, 1.1 Hz, 1H), 6.97 (ddd, *J* = 8.4, 1.1, 0.5 Hz, 1H), 4.56 (ddt, *J* = 10.1, 8.4, 5.0 Hz, 1H), 2.63 – 2.69 (m, 2H), 1.79 – 1.87 (m, 2H), 1.58 – 1.78 (m, 6H), 1.49 (ddd, *J* = 14.0, 7.8, 4.8 Hz, 1H), 1.23 – 1.32 (m, 3H), 1.18 (qt, *J* = 12.4, 3.3 Hz, 1H). ^{13}C NMR (176 MHz, $CDCl_3$) δ 192.86, 161.86, 136.07, 127.09, 121.28, 121.24, 118.13, 75.98, 43.64, 42.77, 33.82, 33.80, 33.15, 26.60, 26.36, 26.27.
HRMS (ESI+) m/z calcd. for $C_{16}H_{21}O_2^+$ [M+H]$^+$ 245.1536 found 245.1536.

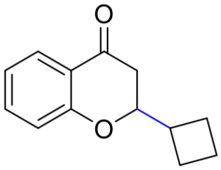

2-cyclobutylchroman-4-one (3k): Synthesized according to general procedure, yielding the desired product as yellow oil, 17 mg, yield: 57 %; Elutes with 5% EtOAc in petroleum ether. ^{1}H NMR (700 MHz, $CDCl_3$) δ 7.87 (dd, *J* = 8.3, 1.8 Hz, 1H), 7.44 – 7.51 (m, 1H), 6.97 – 7.01 (m, 2H), 4.37 (ddd, *J* = 11.4, 7.2, 3.9 Hz, 1H), 2.61 (dd, *J* = 16.7, 3.9 Hz, 1H), 2.57 (dd, *J* = 16.7, 11.7 Hz, 1H), 2.12 – 2.19 (m, 1H), 2.04 – 2.11 (m, 2H), 1.85 – 2.03 (m, 4H). ^{13}C NMR (176 MHz, $CDCl_3$) δ 192.86, 161.94, 136.08, 127.07, 121.23, 121.19, 118.12, 80.97, 40.52, 39.08, 24.34, 23.51, 18.29.
HRMS (ESI+) m/z calcd. for $C_{13}H_{15}O_2^+$ [M+H]$^+$ 203.1066 found 203.1067.

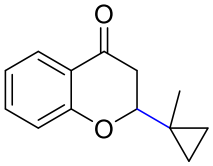

2-(1-methylcyclopropyl)chroman-4-one (3l): Synthesized according to general procedure, yielding the desired product as yellow oil, 16 mg, yield: 53 %; Elutes with 5% EtOAc in petroleum ether. ^{1}H NMR (700 MHz, $CDCl_3$) δ 7.87 (ddd, *J* = 7.8, 2.0, 0.6 Hz, 1H), 7.47 (ddd, *J* = 8.4, 7.1, 1.8 Hz, 1H), 6.97 – 7.03 (m, 2H), 3.74 (dd, *J* = 14.1, 2.4 Hz, 1H), 2.89 (dd, *J* = 16.6, 14.1 Hz, 1H), 2.64 (dd, *J* = 16.6, 2.4 Hz, 1H), 1.21 (s, 3H), 0.65 – 0.70 (m, 1H), 0.56

(ddd, J = 8.8, 5.7, 4.5 Hz, 1H), 0.52 (dt, J = 9.9, 5.1 Hz, 1H), 0.44 (ddd, J = 9.0, 5.9, 4.6 Hz, 1H). ^{13}C NMR (176 MHz, $CDCl_3$) δ 193.37, 162.26, 134.81, 127.08, 121.25, 121.00, 118.12, 84.44, 40.81, 18.60, 18.31, 12.79, 10.29.

HRMS (ESI+) m/z calcd. for $C_{13}H_{15}O_2^+$ [M+H]$^+$ 203.1066 found 203.1067.

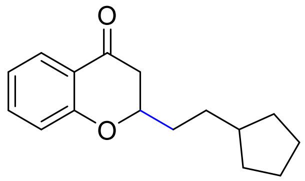

2-(2-cyclopentylethyl)chroman-4-one (3m): Synthesized according to general procedure, yielding the desired product as yellow oil, 16 mg, yield: 44 %; Elutes with 5% EtOAc in petroleum ether.

^{1}H NMR (700 MHz, $CDCl_3$) δ 7.87 (dd, J = 7.8, 1.8 Hz, 1H), 7.47 (ddd, J = 8.4, 7.2, 1.8 Hz, 1H), 6.95 – 7.04 (m, 2H), 4.43 (qd, J = 7.6, 5.2 Hz, 1H), 2.65 – 2.74 (m, 2H), 1.86 – 1.94 (m, 1H), 1.49 – 1.69 (m, 10H), 1.09 – 1.14 (m, 2H). ^{13}C NMR (176 MHz, $CDCl_3$) δ 192.85, 161.87, 136.08, 127.09, 121.27, 121.20, 118.07, 78.30, 43.18, 40.05, 32.84, 32.79, 32.44, 31.39, 25.33.

HRMS (ESI+) m/z calcd. for $C_{16}H_{21}O_2^+$ [M+H]$^+$ 245.1536 found 245.1536.

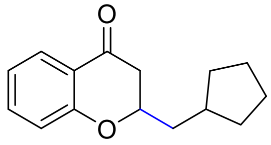

2-(cyclopentylmethyl)chroman-4-one (3n): Synthesized according to general procedure, yielding the desired product as yellow oil, 19 mg, yield: 56 %; Elutes with 5% EtOAc in petroleum ether.

^{1}H NMR (400 MHz, $CDCl_3$) δ 7.88 (dd, J = 7.9, 1.9 Hz, 1H), 7.47 (ddd, J = 8.3, 7.2, 1.8 Hz, 1H), 6.92 – 7.04 (m, 2H), 4.42 – 4.54 (m, 1H), 2.62 – 2.76 (m, 2H), 2.03 – 2.11 (m, 1H), 1.97 (ddd, J = 13.7, 7.9, 6.7 Hz, 1H), 1.78 – 1.91 (m, 2H), 1.57 – 1.71 (m, 4H), 1.51 – 1.56 (m, 1H), 1.14 – 1.17 (m, 2H). ^{13}C NMR (176 MHz, $CDCl_3$) δ 192.82, 161.84, 136.08, 127.09, 121.26, 121.22, 118.11, 77.69, 43.51, 41.33, 36.29, 33.07, 32.84, 25.24, 25.06.

HRMS (ESI+) m/z calcd. for $C_{15}H_{19}O_2^+$ [M+H]$^+$ 231.1379 found 231.1380.

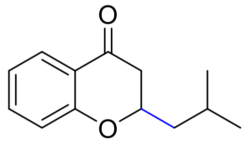

2-isobutylchroman-4-one (3o): Synthesized according to general procedure, yielding the desired product as yellow oil, 16 mg, yield: 53 %; Elutes with 5% EtOAc in petroleum ether.

^{1}H NMR (700 MHz, $CDCl_3$) δ 7.88 (ddd, J = 7.8, 1.8, 0.5 Hz, 1H), 7.47 (ddd, J = 8.3, 7.2, 1.8 Hz, 1H), 7.00 (ddd, J = 8.0, 7.1, 1.1 Hz, 1H), 6.97 (dd, J = 8.5, 1.0 Hz, 1H), 4.53 (ddt, J = 10.0, 8.6, 5.0 Hz, 1H), 2.63 – 2.71 (m, 2H), 1.77 – 1.92 (m, 2H), 1.44 – 1.47 (m, 1H), 0.97 – 0.98 (m, 6H). ^{13}C NMR (176 MHz, $CDCl_3$):δ 192.9, 161.8, 136.0, 127.1, 121.3, 121.2, 118.1, 76.4, 44.1, 43.6, 24.4, 23.7, 23.1.

HRMS (ESI+) m/z calcd. for $C_{13}H_{17}O_2^+$ [M+H]$^+$ 205.1223 found 205.1223.

The characteristics data is consistent with the one reported before.[5]

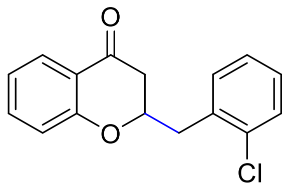

2-(2-chlorobenzyl)chroman-4-one (3p): Synthesized according to general procedure, yielding the desired product as yellow oil, 21 mg, yield: 52 %; Elutes with 5% EtOAc in petroleum ether.

^{1}H NMR (700 MHz, $CDCl_3$) δ 7.87 (ddd, J = 7.8, 1.8, 0.5 Hz, 1H), 7.47 (ddd, J = 8.4, 7.2, 1.8 Hz, 1H), 7.39 (dd, J = 7.5, 1.8 Hz, 1H), 7.32 (dd, J = 7.3, 2.1 Hz, 1H), 7.21 – 7.25 (m, 2H), 7.01 (ddd, J = 8.0, 7.1, 1.1 Hz, 1H), 6.96 (dd, J = 8.4, 1.5 Hz, 1H), 4.73 – 4.82 (m, 1H), 3.36 (dd, J = 13.9, 6.9 Hz, 1H), 3.20 (dd, J = 13.9, 6.2 Hz, 1H), 2.71 – 2.74 (m, 2H). ^{13}C NMR (176 MHz, MeOD) δ 194.10, 162.93, 137.46, 135.74, 135.40, 133.26, 130.63, 129.66, 128.14, 127.64, 122.46, 122.11, 119.12, 78.39, 43.20, 39.28. HRMS (ESI+) m/z calcd. for $C_{16}H_{13}ClO_2^+$ [M+H] 273.06767 found 273.0677.

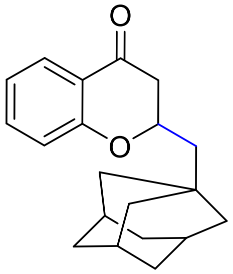

2-(((1r,3*R*,5*S*)-adamantan-1-yl)methyl)chroman-4-one (3r): Synthesized according to general procedure, yielding the desired product as yellow oil, 17 mg, yield: 39 %; Elutes with 5% EtOAc in petroleum ether.

^{1}H NMR (700 MHz, $CDCl_3$) δ 7.86 (dd, *J* = 8.1, 1.7 Hz, 1H), 7.50 – 7.43 (m, 1H), 7.02 – 6.95 (m, 2H), 3.91 (dd, *J* = 14.2, 2.4 Hz, 1H), 2.72 (dd, *J* = 16.4, 14.2 Hz, 1H), 2.62 (dd, *J* = 16.5, 2.5 Hz, 1H), 2.06 (s, 3H), 1.77 (d, *J* = 12.1 Hz, 6H), 1.71 (d, *J* = 11.4 Hz, 4H), 1.67 – 1.62 (m, 3H), 1.55 (s, 1H).

^{13}C NMR (176 MHz, $CDCl_3$) δ 193.03, 161.76, 136.05, 127.05, 124.06, 121.27, 118.24, 74.79, 49.38, 44.91, 42.93, 37.11, 32.38, 28.78. HRMS (ESI+) m/z calcd. for $C_{20}H_{24}O_2^+$ [M+H] 297.18489 found 297.1849.

Reference

3. M. Moczulski, E. Kowalska, E. Kuśmierek, Ł. Albrecht, A. Albrecht, *RSC Advances* **2021**, *11*, 27782-27786.
4. S. Li, L. Zhang, Q. He, X. Zhang, C. Yang, *Org. Biomol. Chem*. **2021**, *19*, 5348-5352.
5. R. Chen, J. T. Yu, J. Cheng, *Org. Biomol. Chem*. **2018**, *16*, 3568-3571.

4. NMR Spectra

2-Cyclohexylchroman-4-one 5aa

^{1}H NMR

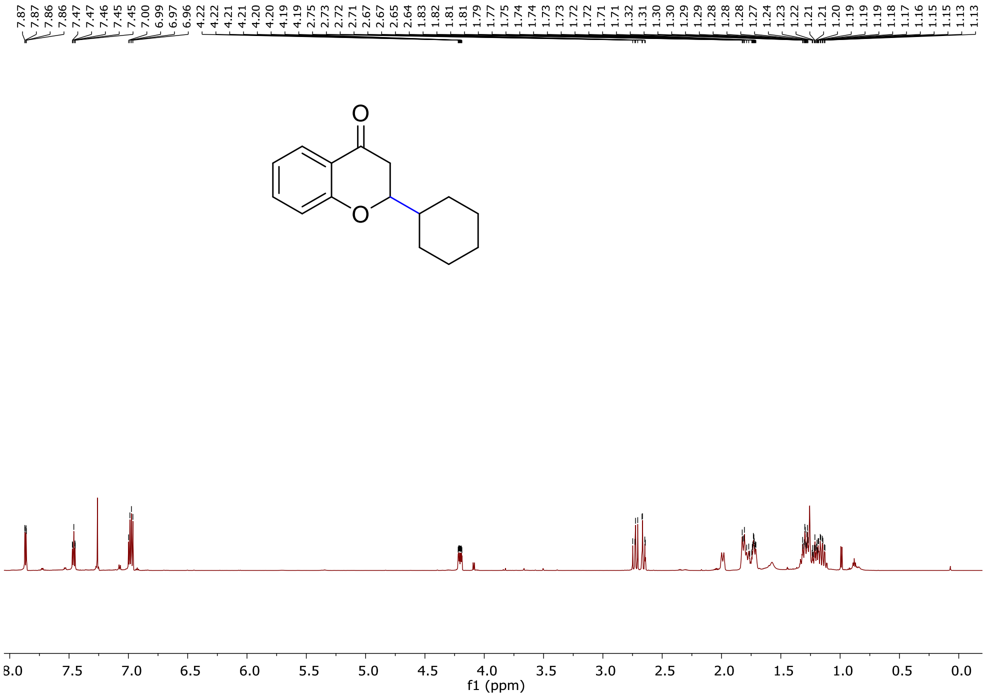

^{13}C NMR

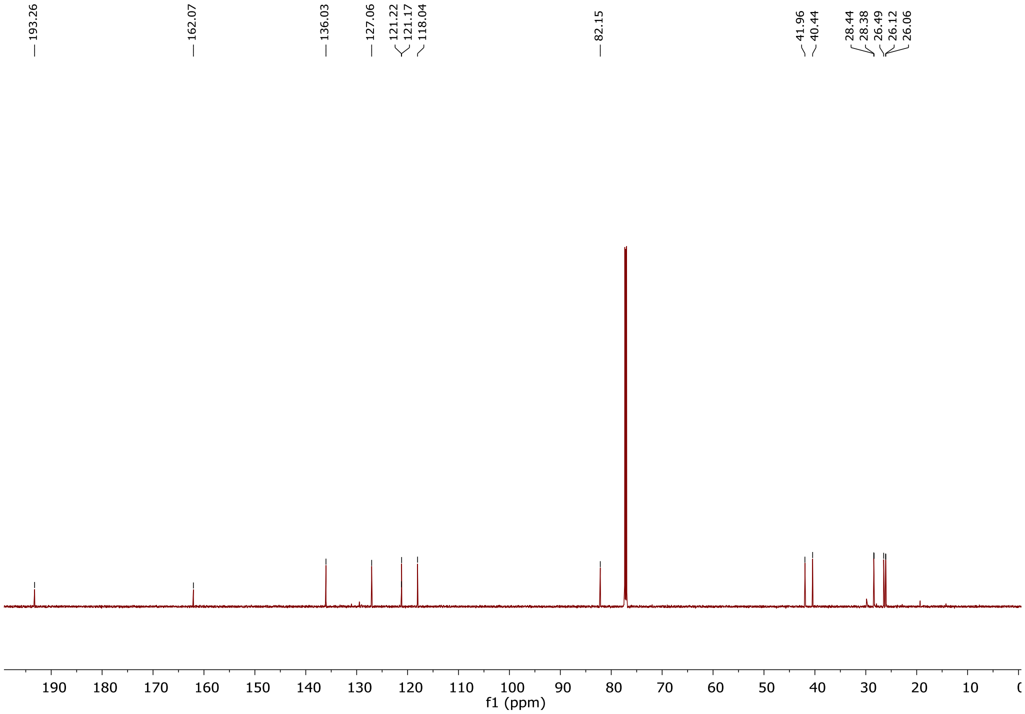

2-cyclohexyl-6-methylchroman-4-one (3b)

^{1}H NMR

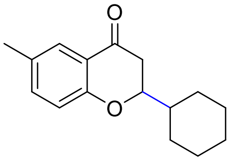

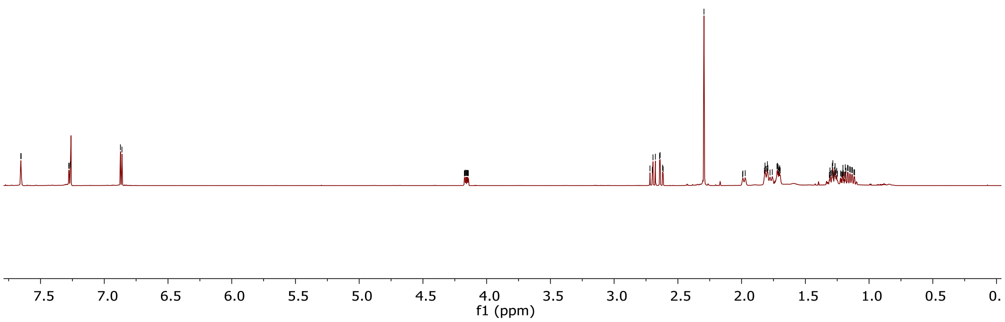

^{13}C NMR

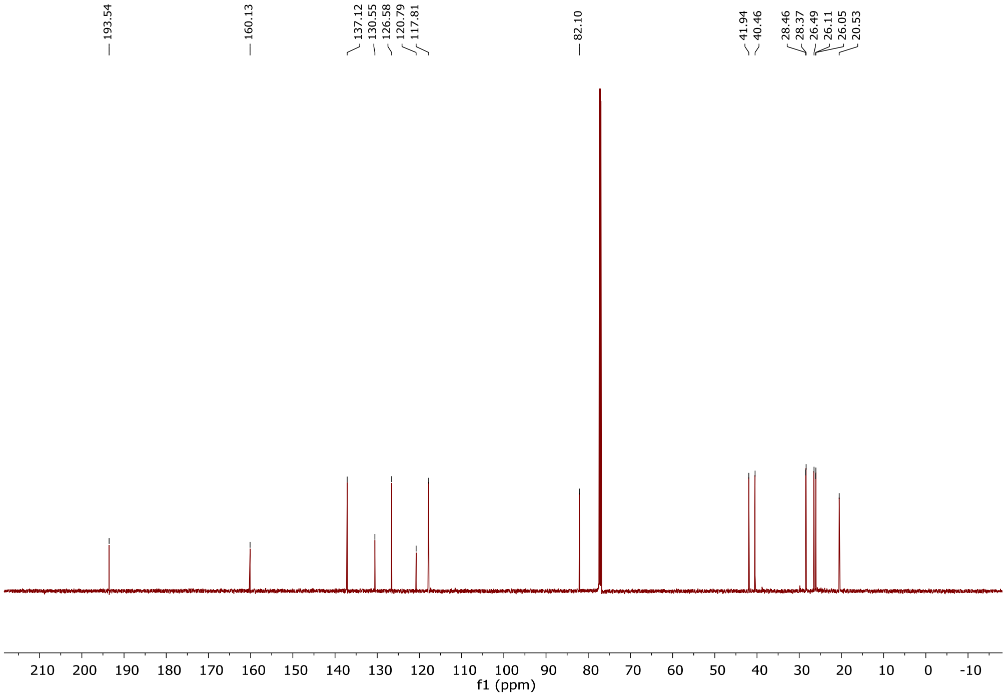

2-cyclohexyl-7-methylchroman-4-one (3c)

^{1}H NMR

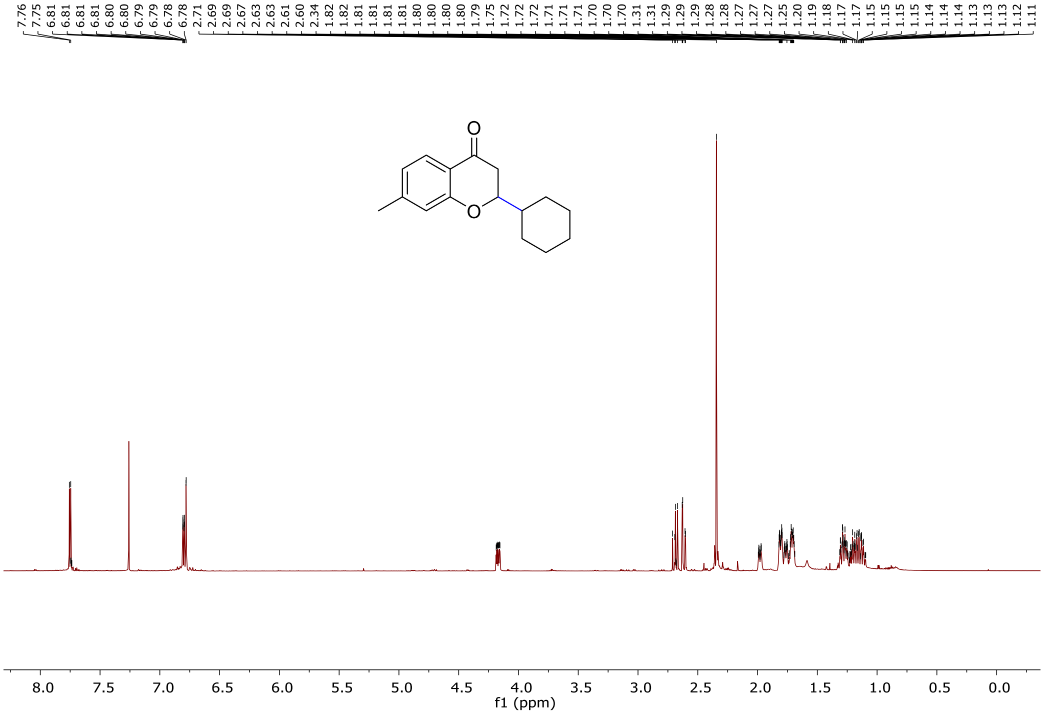

^{13}C NMR

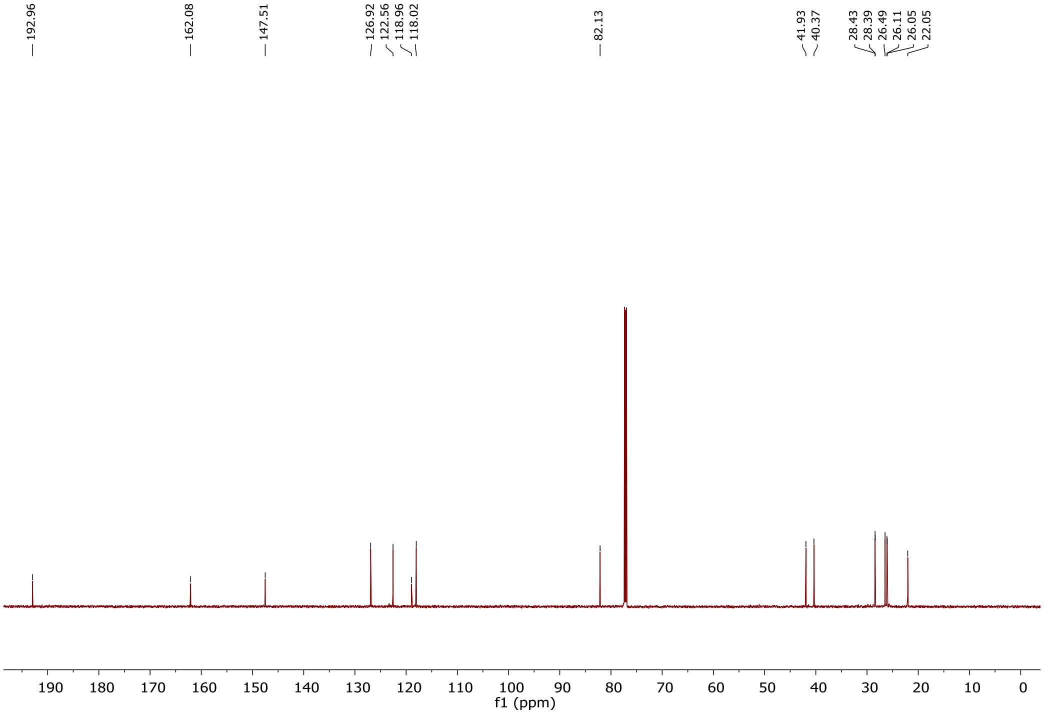

2-cyclohexyl-6-methoxychroman-4-one (3d):

^{1}H NMR

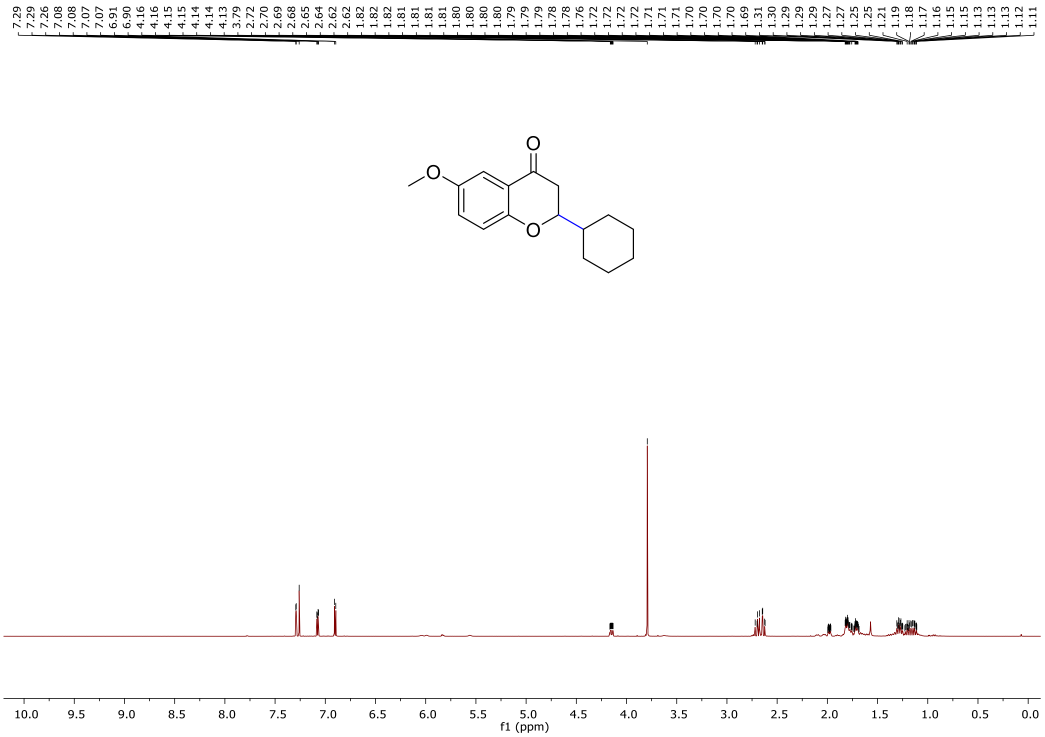

^{13}C NMR

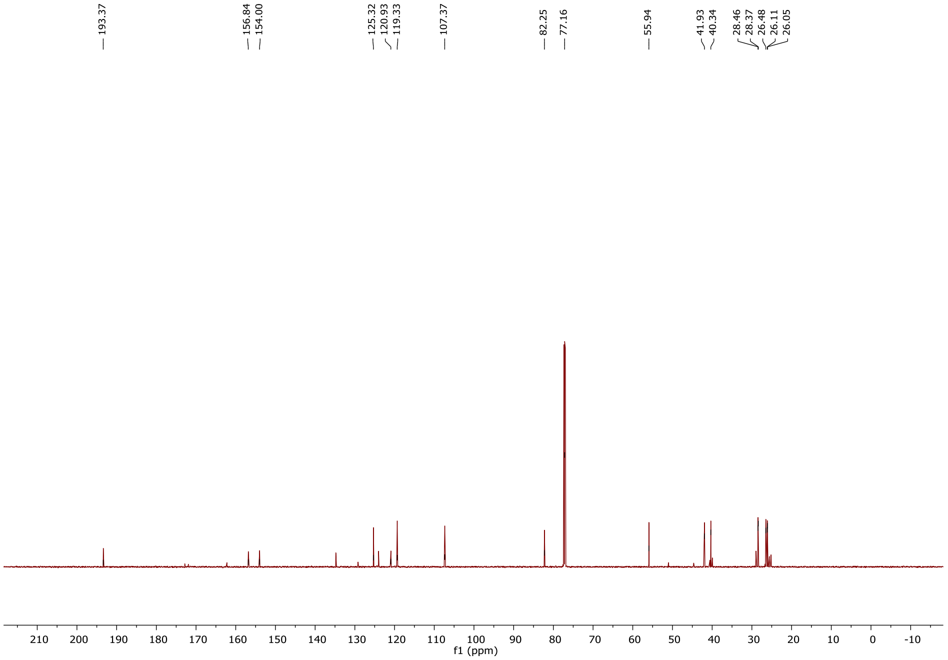

2-cyclohexyl-6-fluorochroman-4-one (3e):
^{1}H NMR

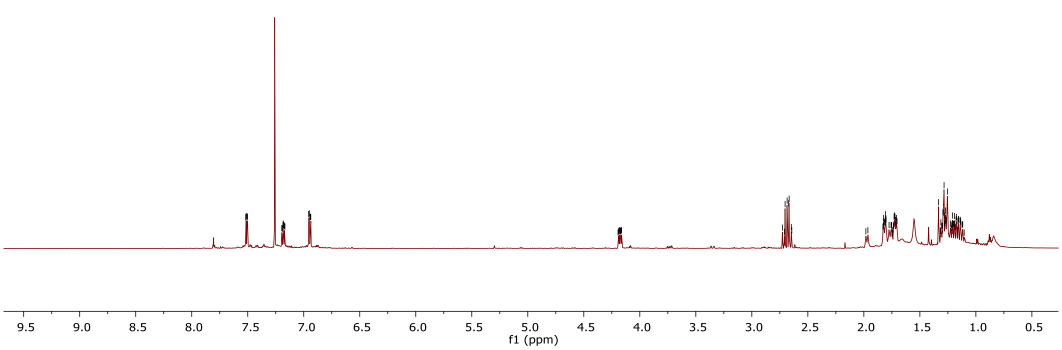

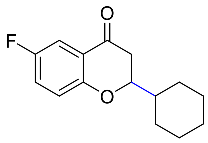

^{13}C NMR

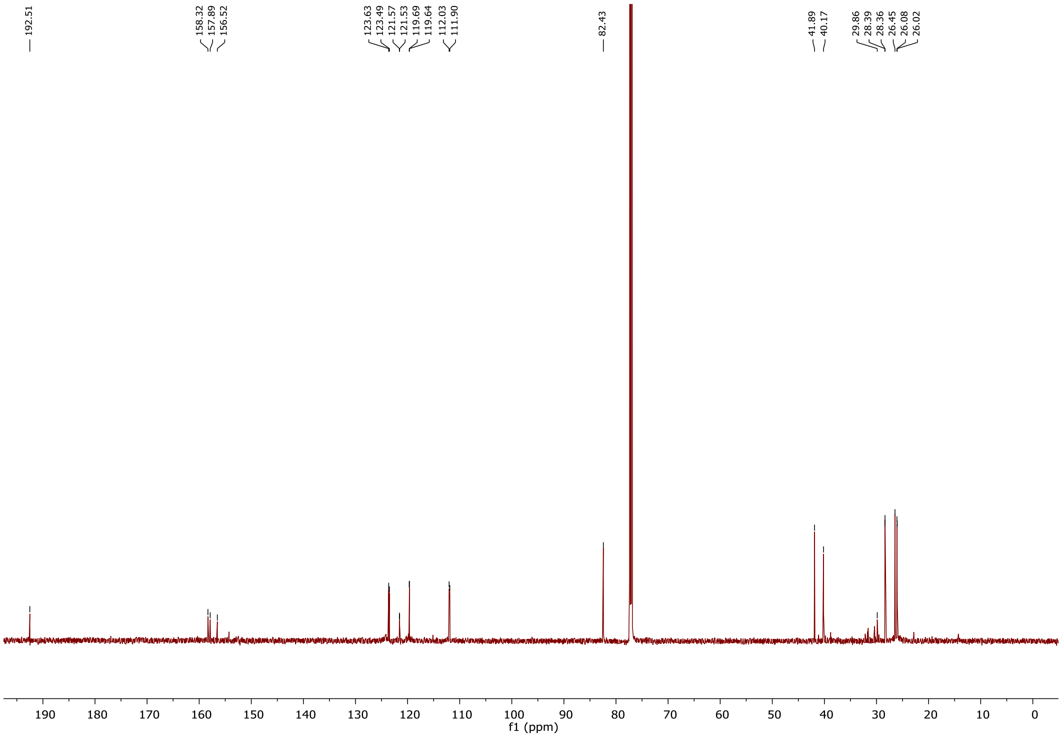

^{19}F NMR

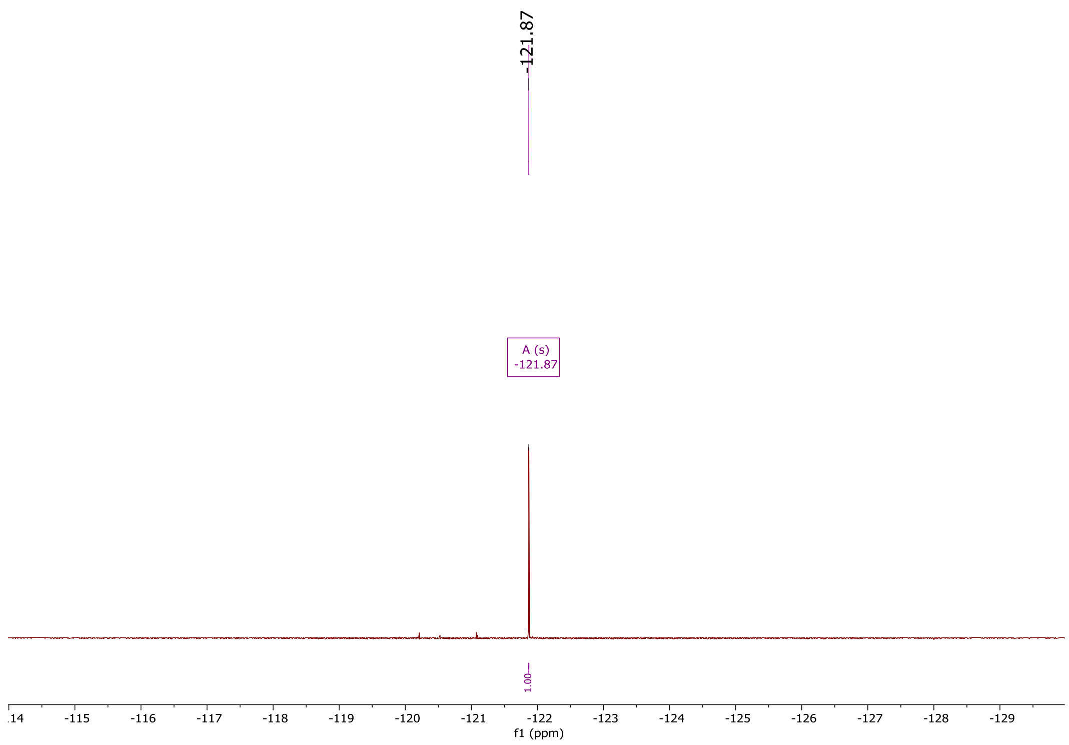

-121.87
A (s)
-121.87
1.00
114
-115
-116
-117
-118
-119
-120
-121
-122
-123
-124
-125
-126
-127
-128
-129
f1 (ppm)

2-cyclohexyl-7-fluorochroman-4-one (3f):

^{1}H NMR

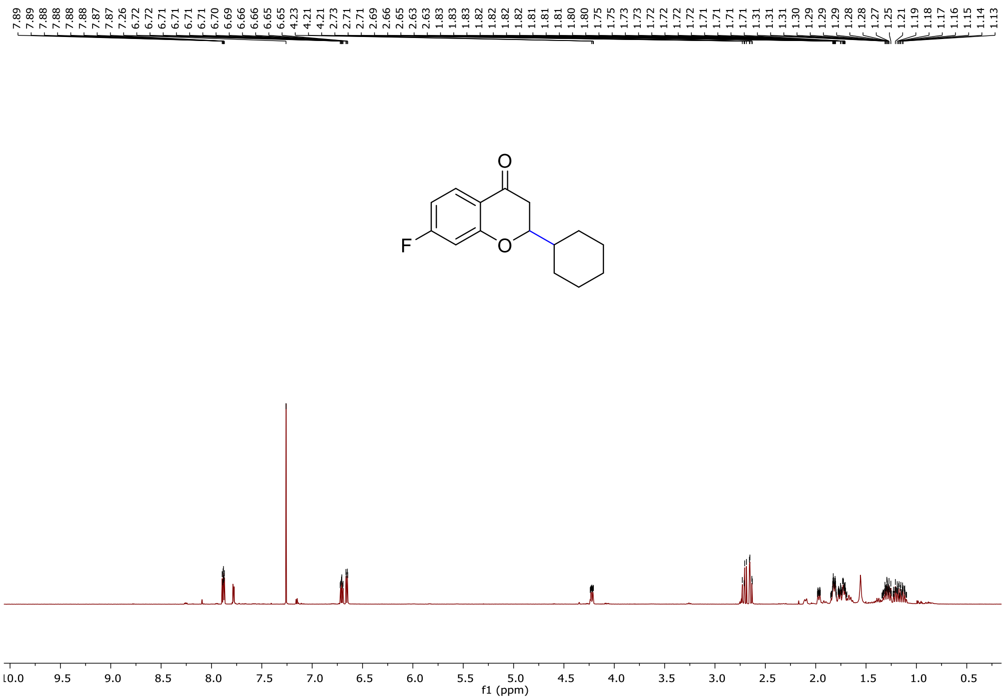

^{13}C NMR

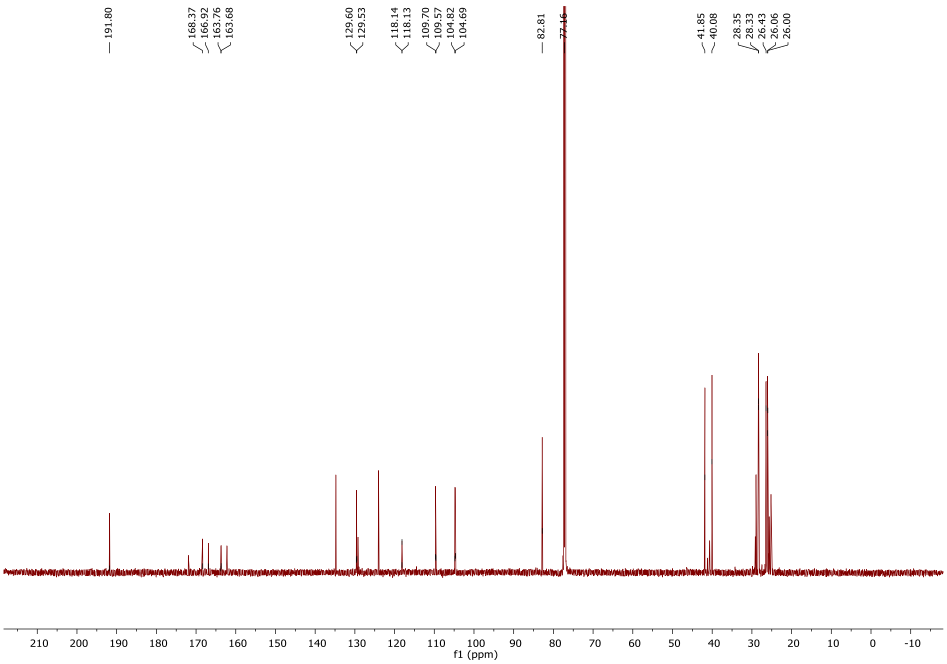

^{19}F NMR

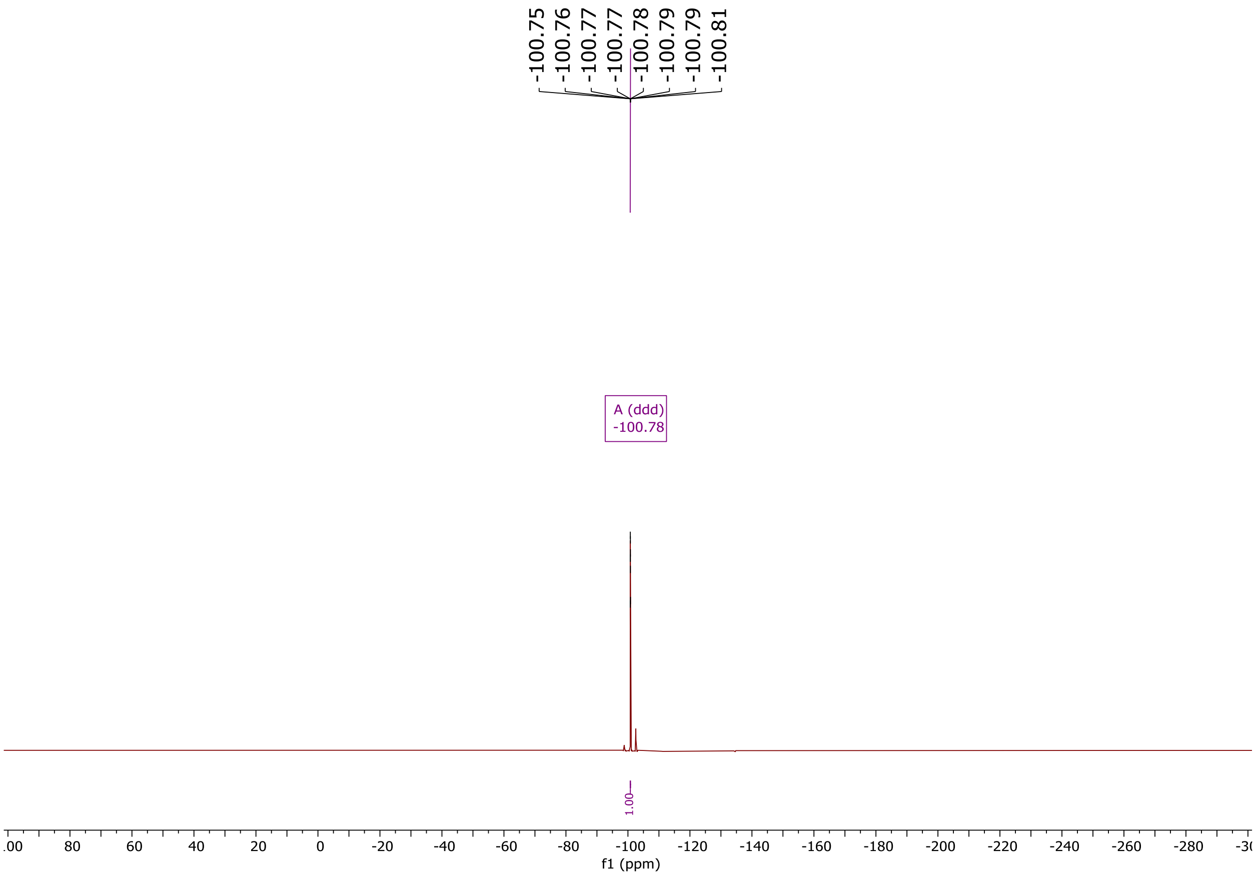
-100.75
-100.76
-100.77
-100.77
-100.78
-100.79
-100.79
-100.81
A (ddd)
-100.78
1.00
f1 (ppm)

6-chloro-2-cyclohexylchroman-4-one (3g):
^{1}H NMR

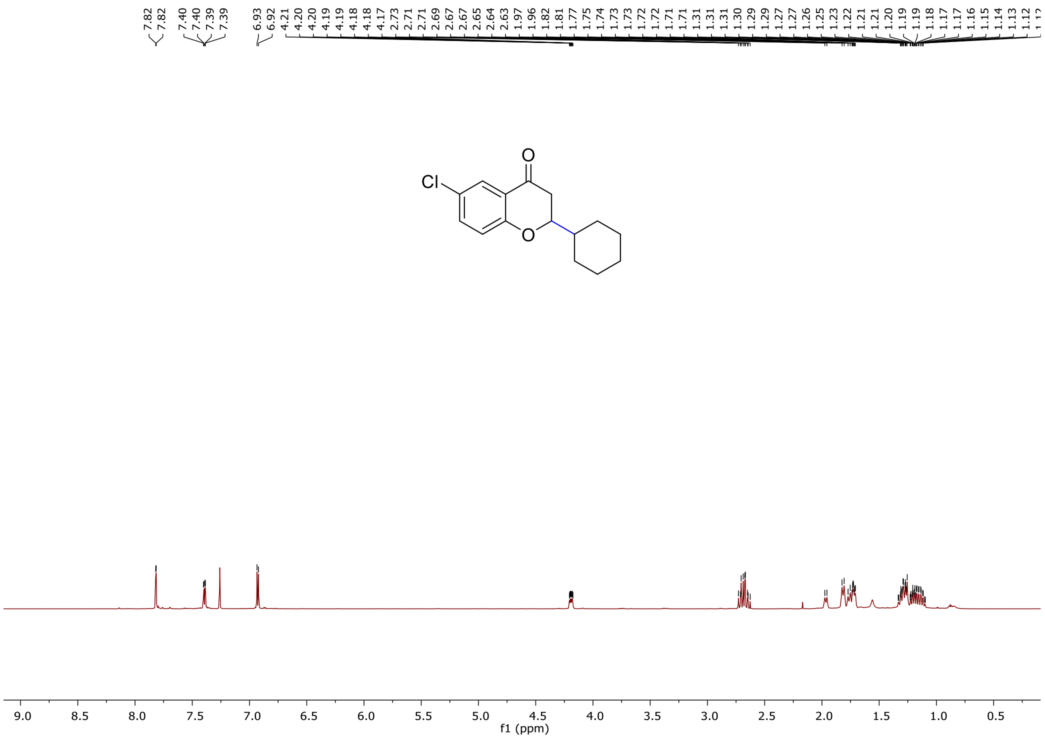

^{13}C NMR

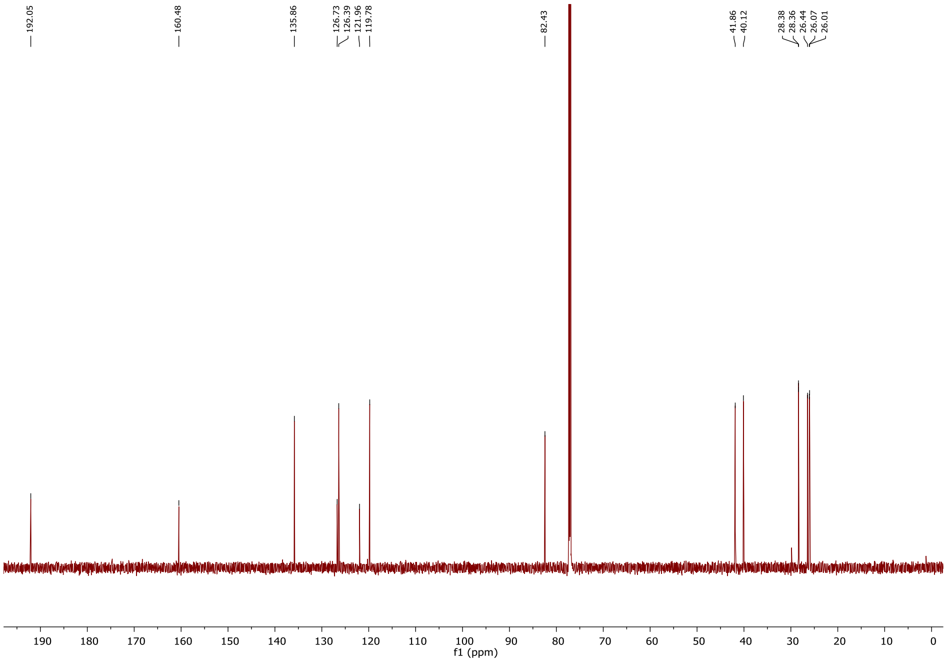

6-bromo-2-cyclohexylchroman-4-one (3h):
^{1}H NMR

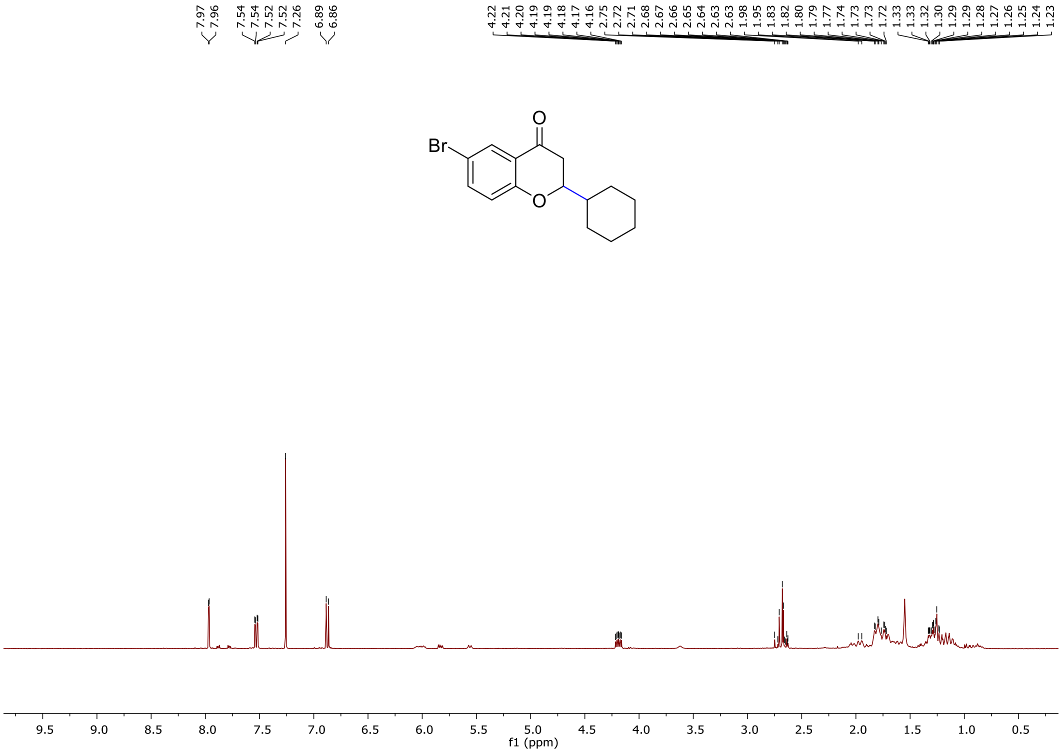

^{13}C NMR

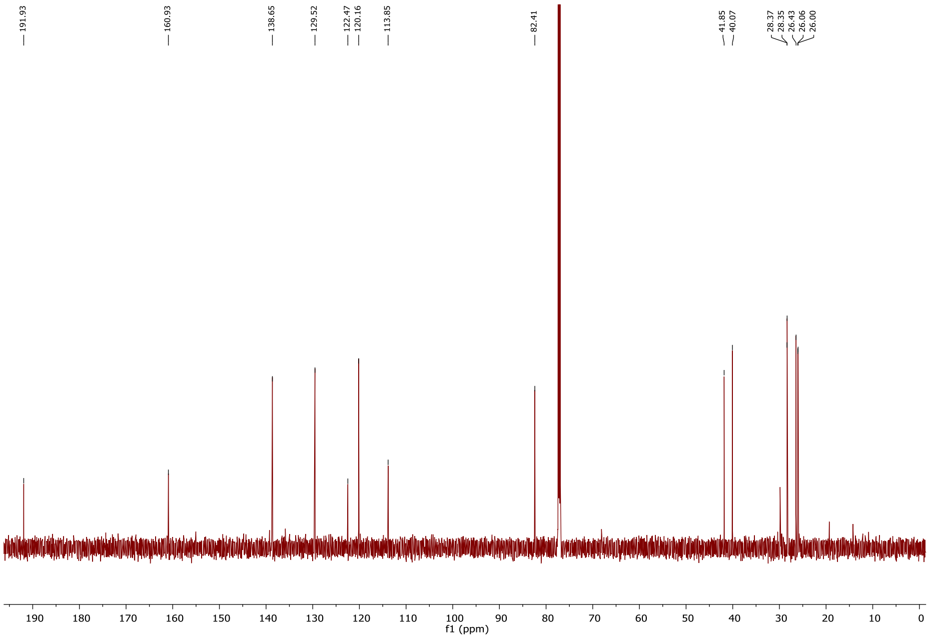

6-chloro-2-cyclohexyl-7-methylchroman-4-one (3i):

^{1}H NMR

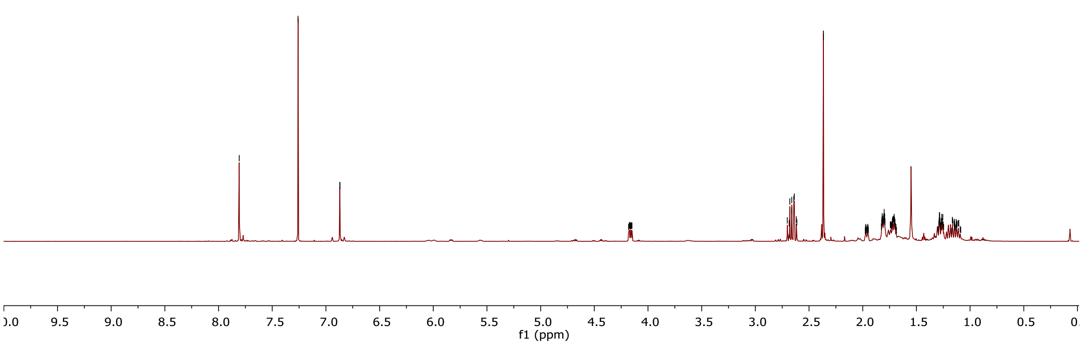

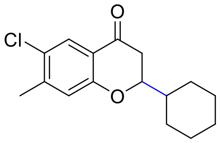

^{13}C NMR

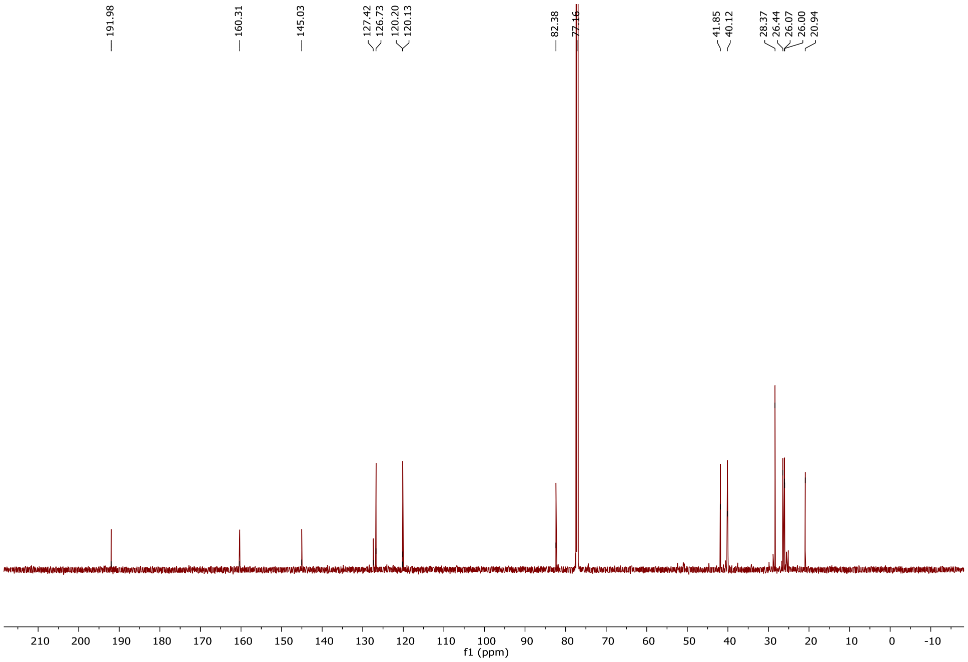

2-(cyclohexylmethyl)chroman-4-one (3j)

^{1}H NMR

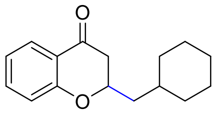

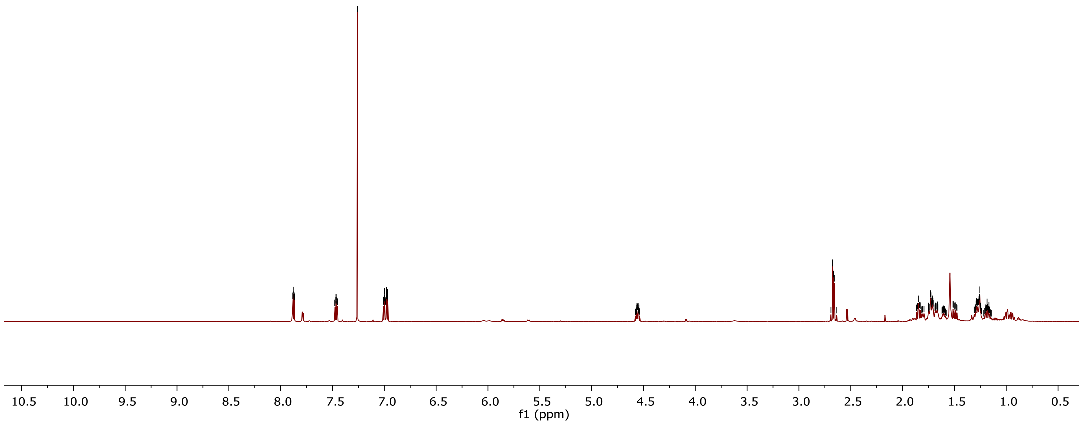

^{13}C NMR

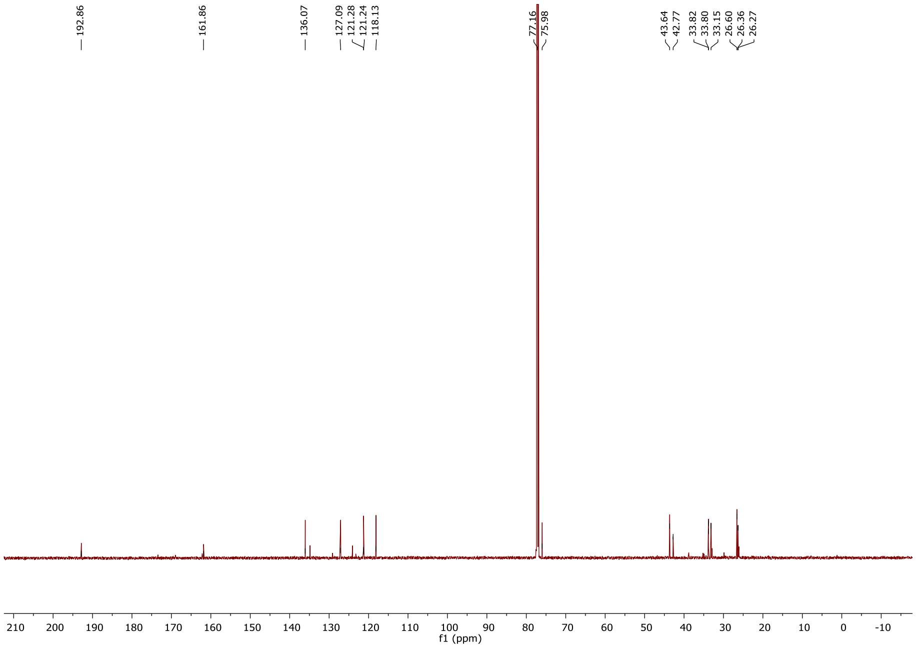

2-cyclobutylchroman-4-one (3k)

^{1}H NMR

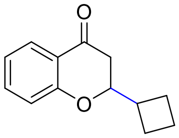

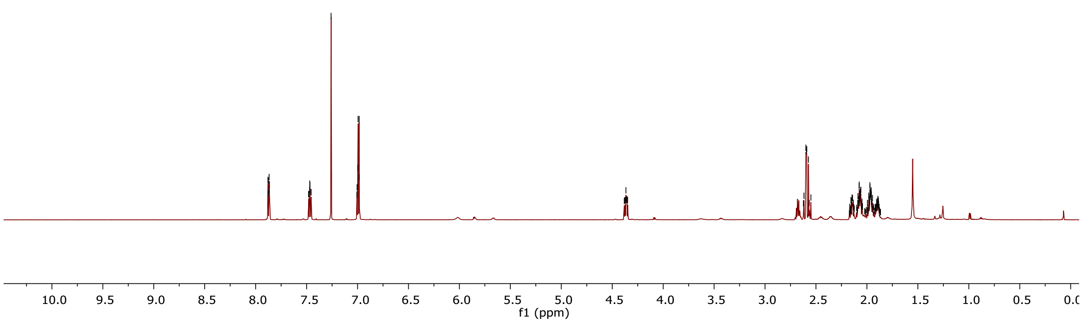

^{13}C NMR

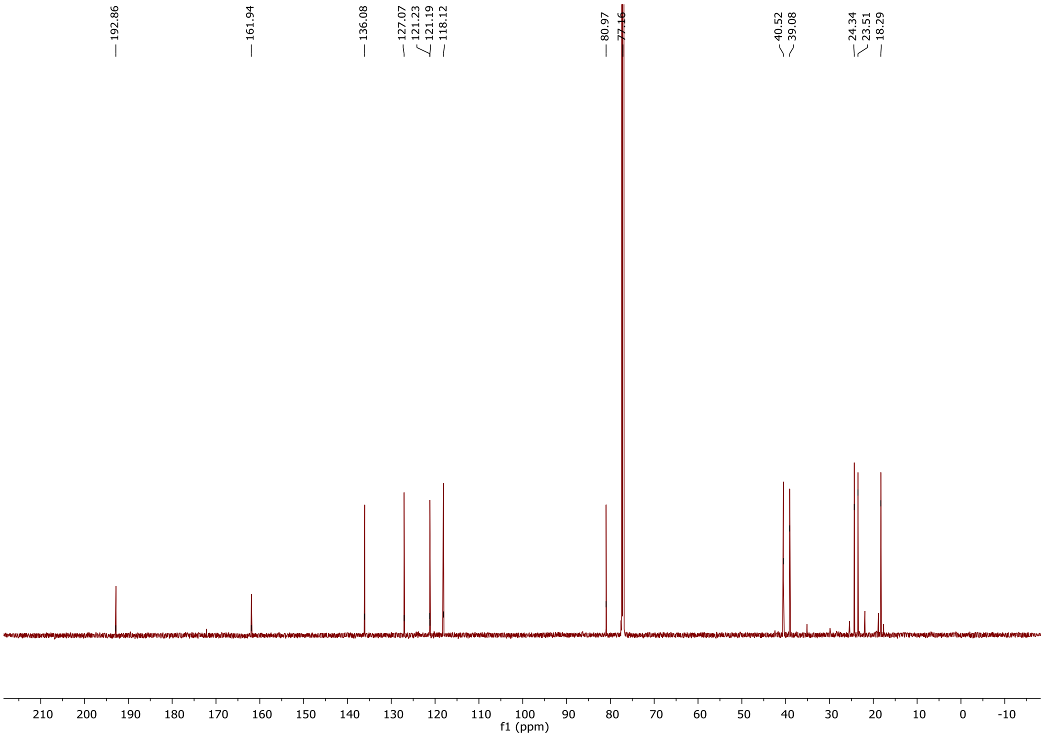

2-(1-methylcyclopropyl)chroman-4-one (3l)

^{1}H NMR

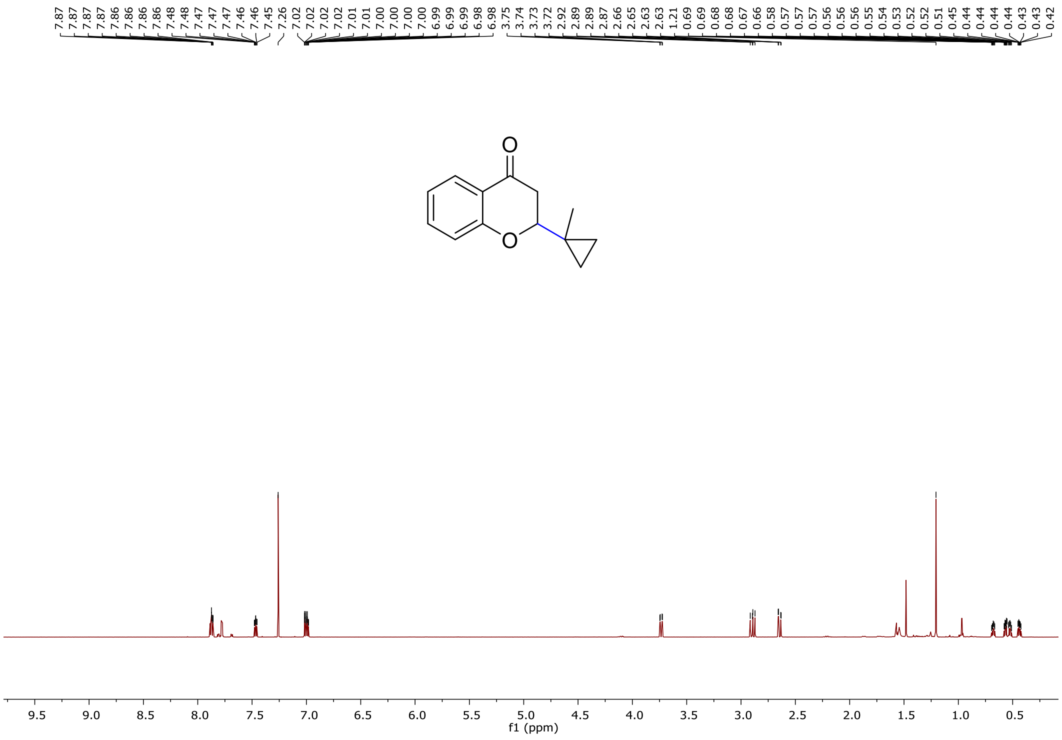

^{13}C NMR

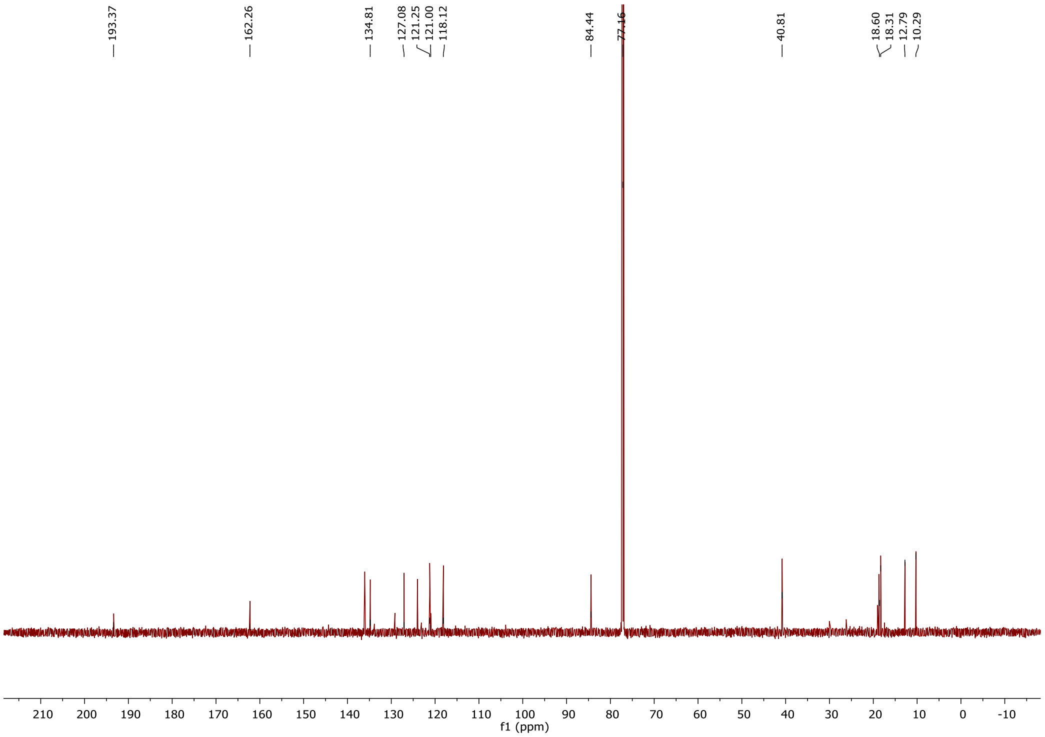

2-(2-cyclopentylethyl)chroman-4-one (3m)

^{1}H NMR

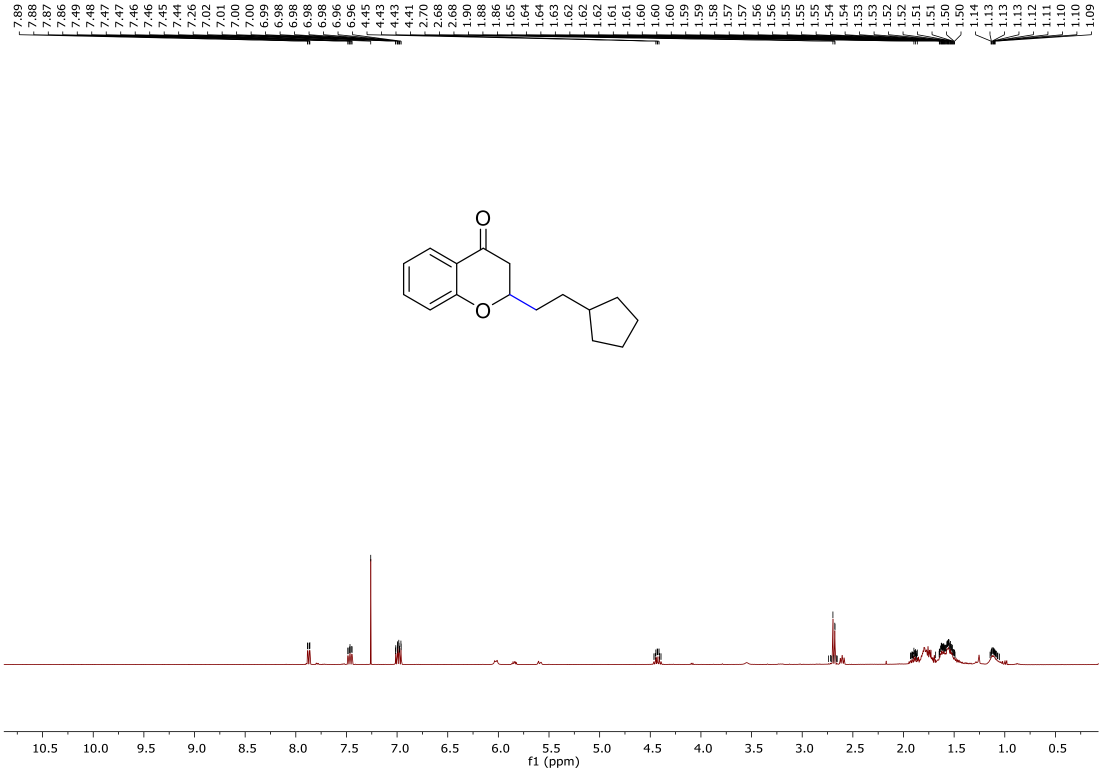

^{13}C NMR

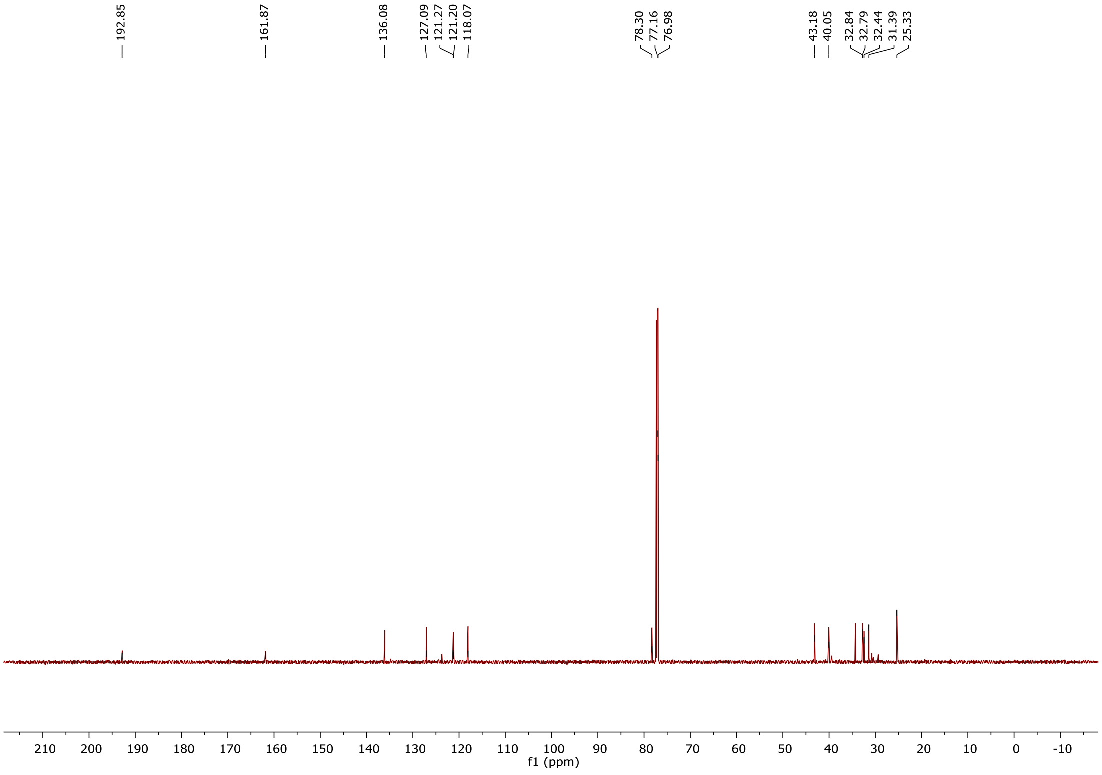

2-(cyclopentylmethyl)chroman-4-one (3n)

^{1}H NMR

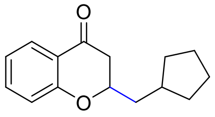

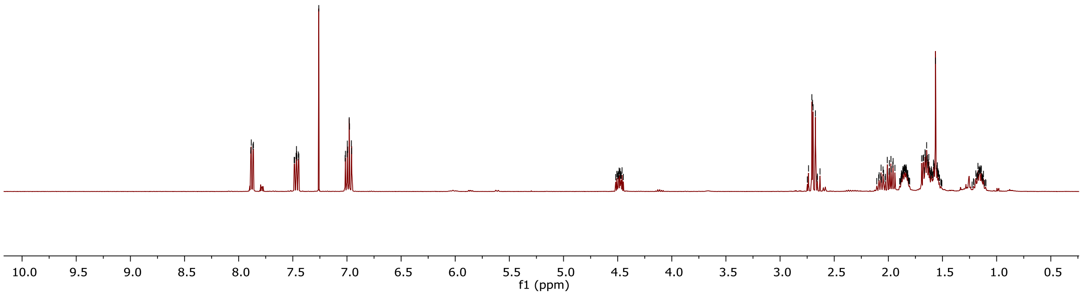

^{13}C NMR

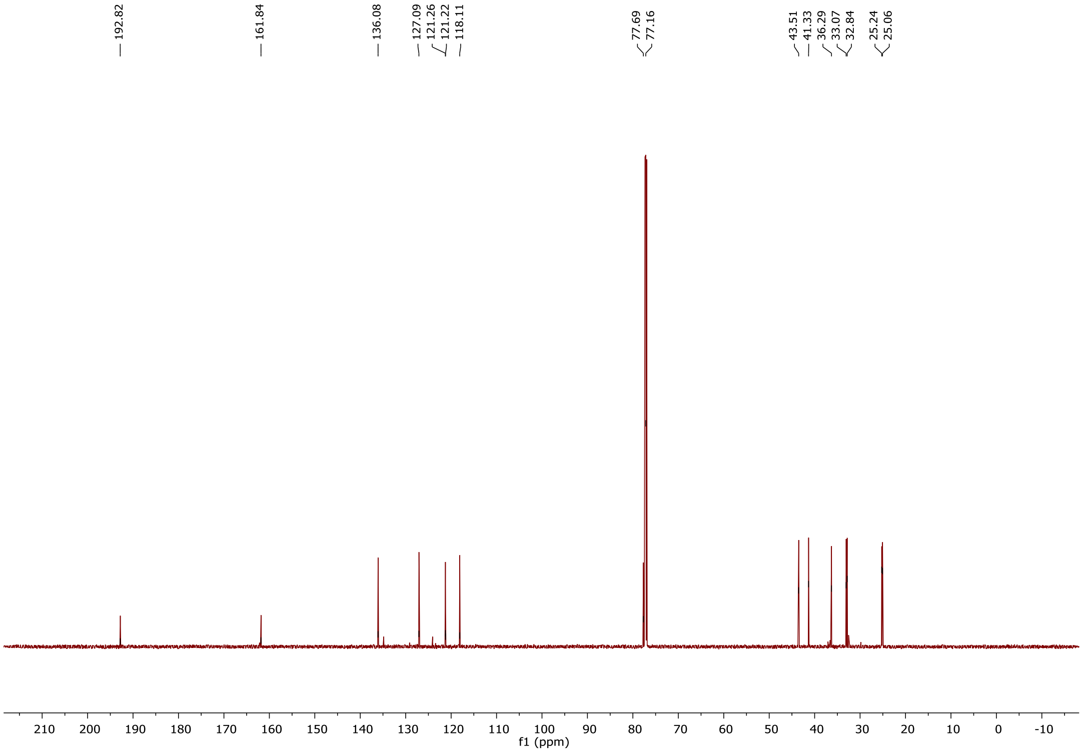

2-isobutylchroman-4-one (3o)

^{1}H NMR

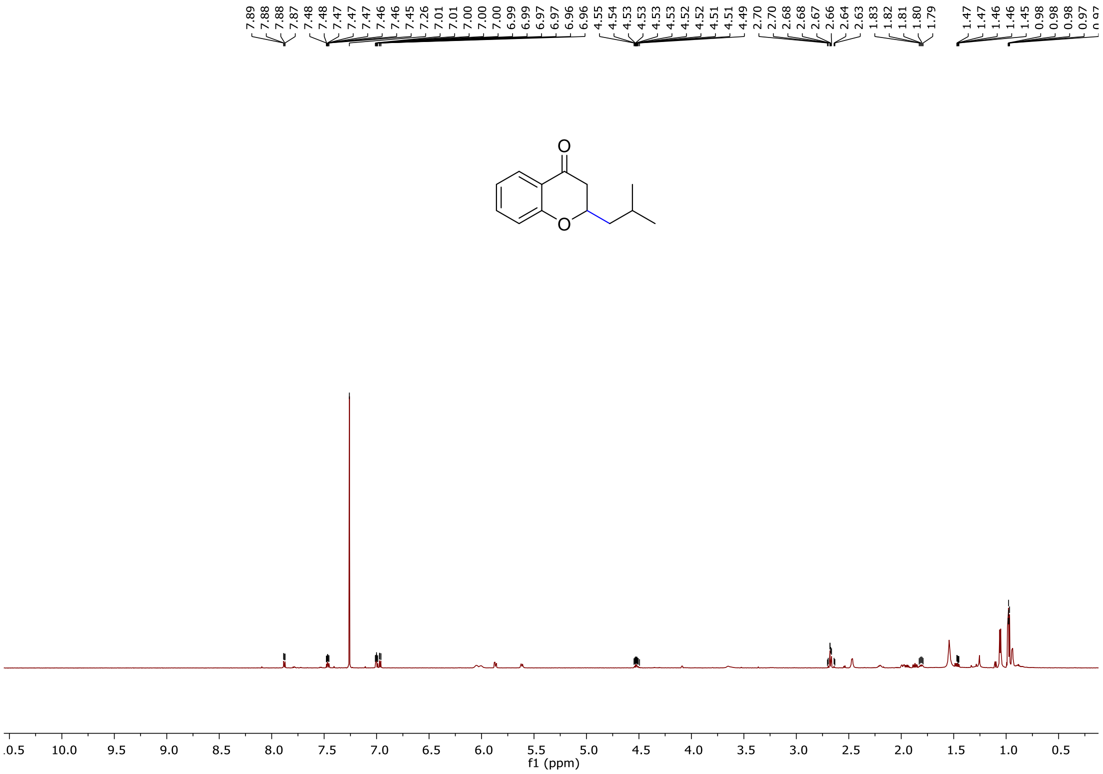

^{13}C NMR

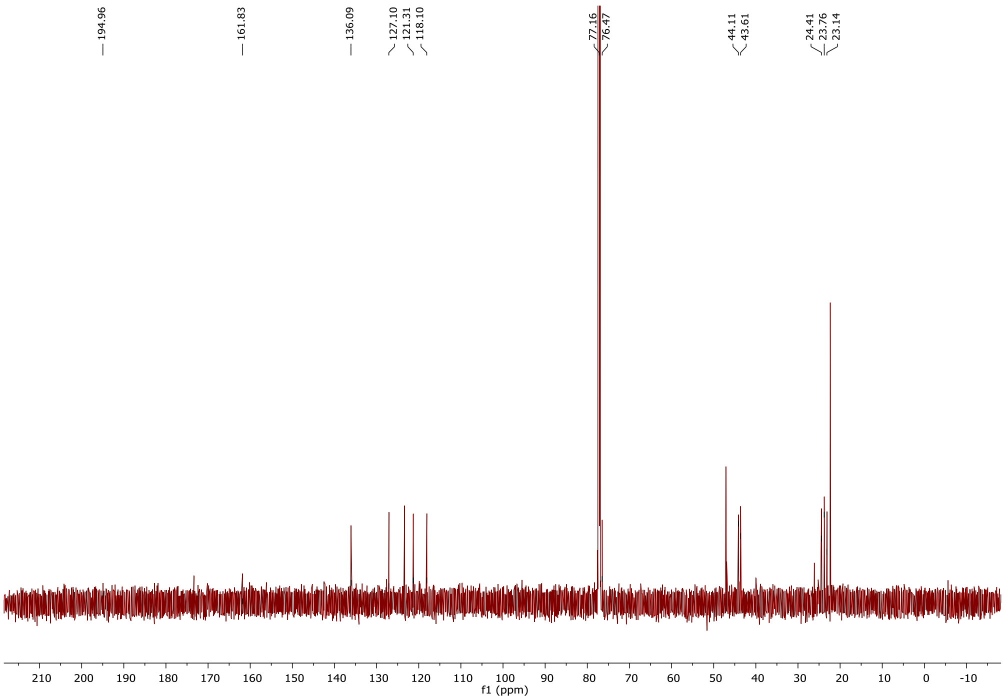

2-(2-chlorobenzyl)chroman-4-one (3p)

^{1}H NMR

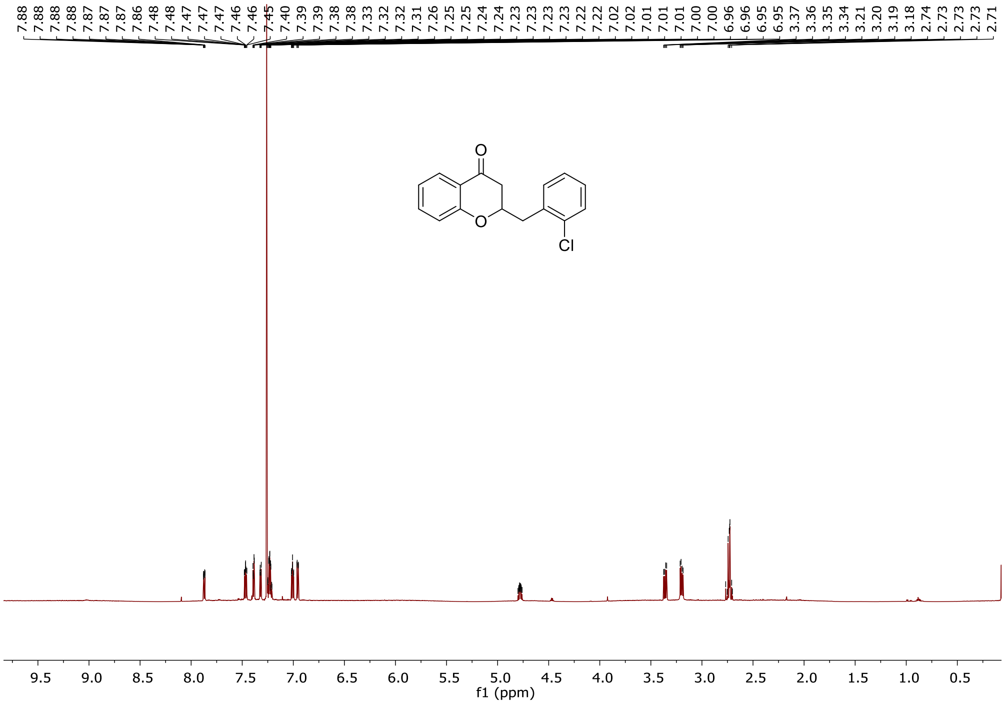

^{13}C NMR

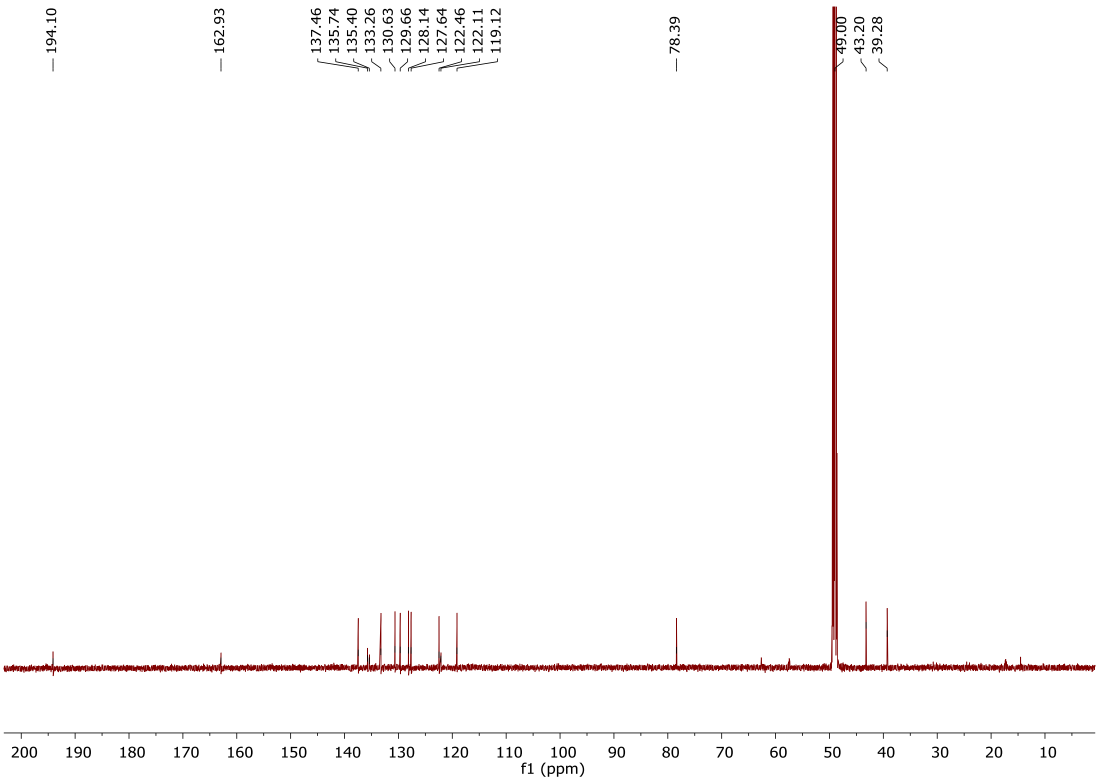

2-(((1r,3*R*,5*S*)-adamantan-1-yl)methyl)chroman-4-one (3r)

^{1}H NMR

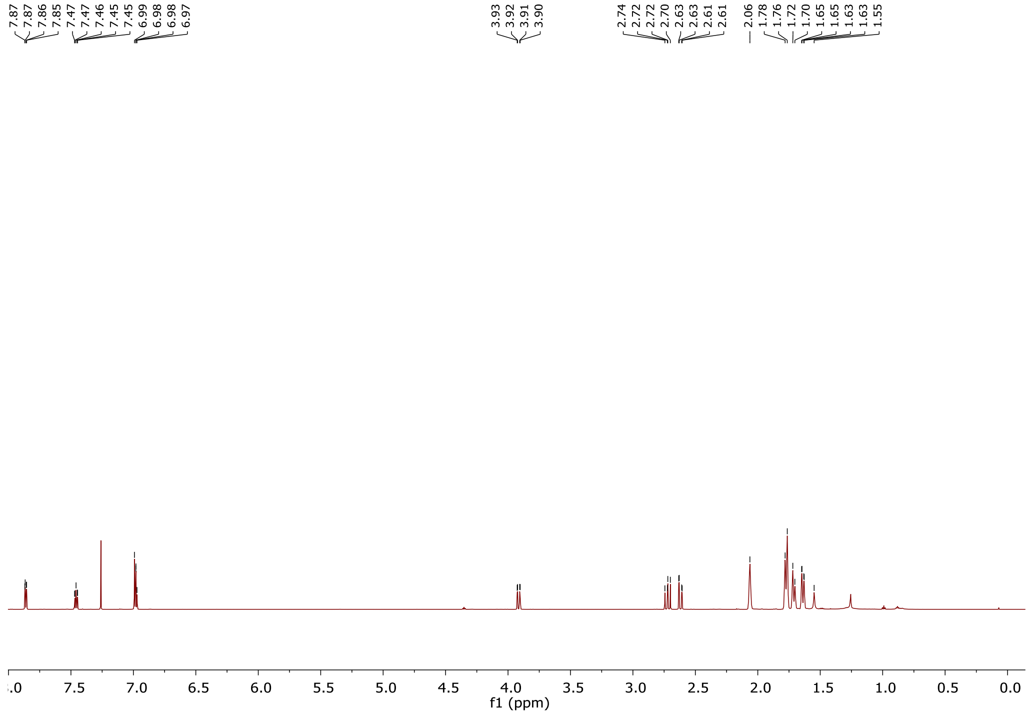

^{13}C NMR

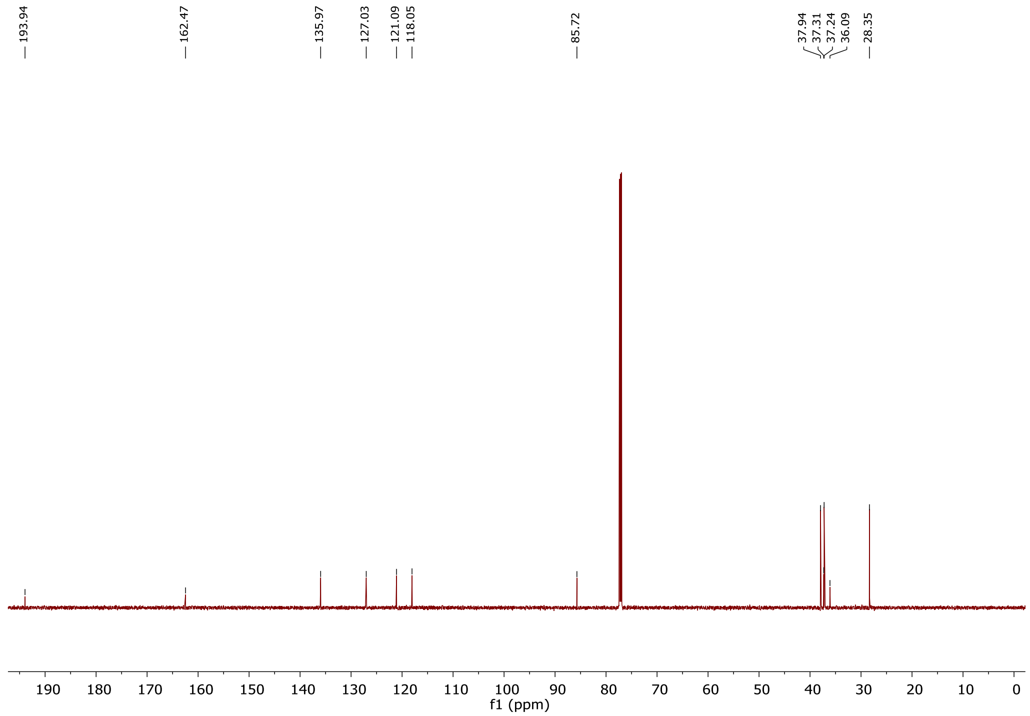

Radical captured experiment by TEMPO

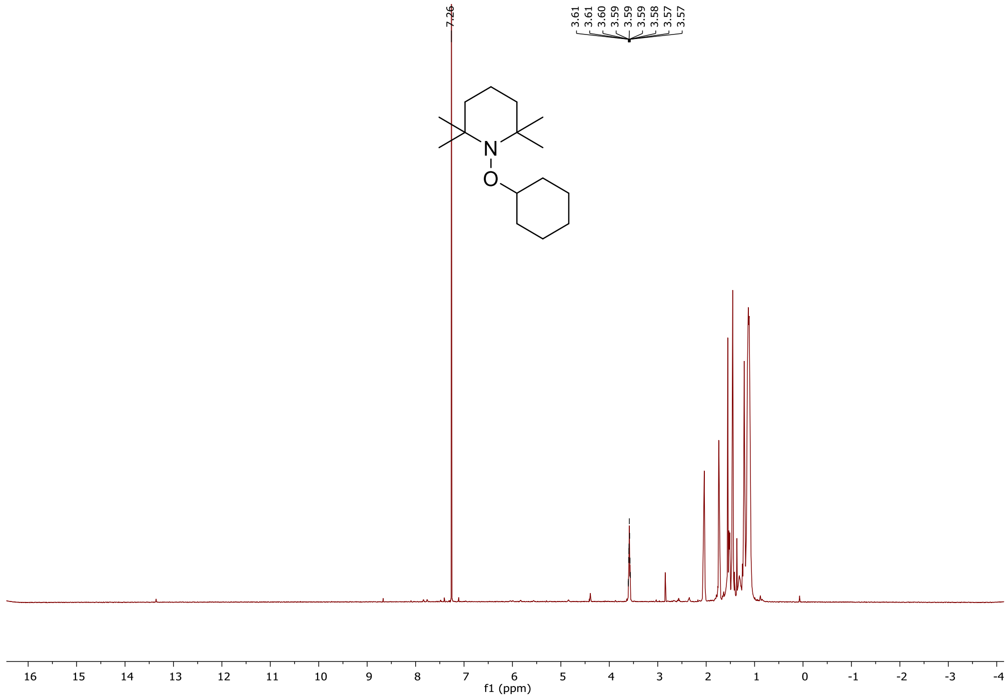

^{13}C NMR

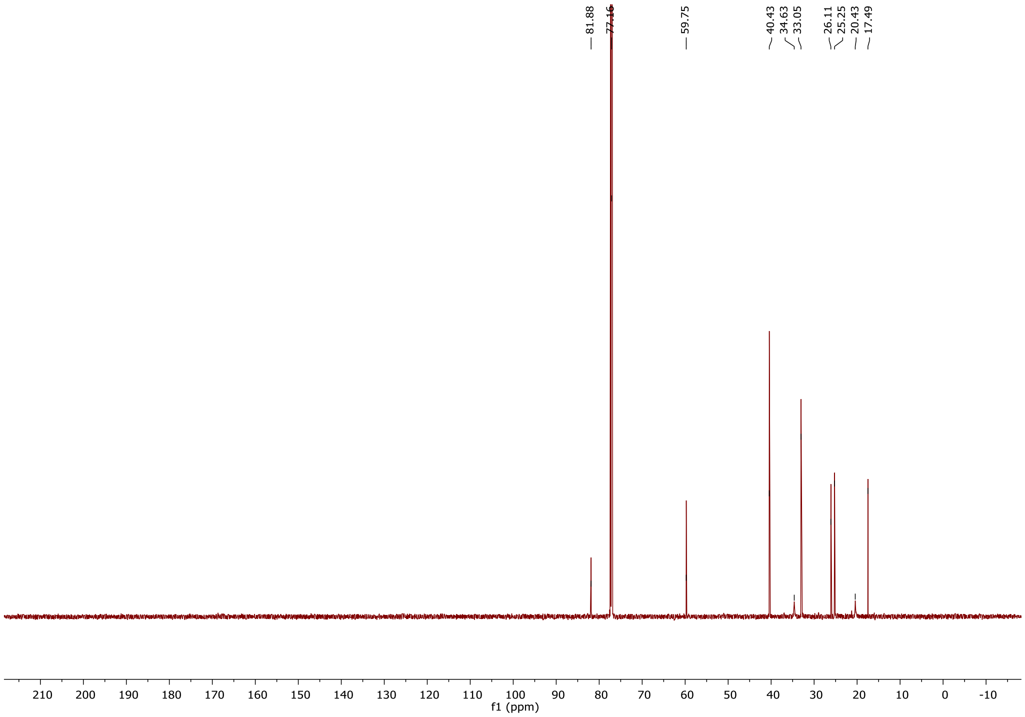

Supplement: Supplementary file 1 — Supporting Information [file OPEN-14-e202400395-s001.pdf]
